# Cocrystal Engineering of Organic Semiconductors for Photovoltaic Applications: Modeling Excited-State Properties of a Charge Transfer Cocrystal of a Dicarbazole Donor and a Fluoranil Acceptor

**DOI:** 10.1021/acs.jpcc.5c06828

**Published:** 2026-01-02

**Authors:** Arkalekha Mandal, Chris Erik Mohn, Carl Henrik Görbitz, Anurag Roy

**Affiliations:** † Department of Chemistry, Blindern Campus, 6305 University of Oslo, Oslo 0371, Norway; ‡ Environmental and Sustainability Institute, Faculty of Environment, Science and Economy, 3286 University of Exeter, Penryn Campus, Exeter, Cornwall TR10 9FE, United Kingdom

## Abstract

With the recent advancements in lightweight, flexible, and environmentally benign organic supramolecular aggregates for various optoelectronic applications, cocrystals of aromatic π-donors and π-acceptors have emerged as promising *n*-type semiconductors and near-infrared absorbers for enhanced photovoltaic properties. Herein, we demonstrate the electron-dominant charge transport and wide absorption spanning from ultraviolet (UV) to NIR-I region (375–800 nm) of a cocrystal with π-donor 4,4′-bis­(carbazol-9-yl)­biphenyl (CBP) and π-acceptor 1,4-tetrafluoro-*p*-benzoquinone (fluoranil) as the components. The crystal packing in CBP:(fluoranil)_2_ is characterized by mixed stacks of alternative CBP and fluoranil molecules tethered by strong *face-to-face* π···π stacking interactions. The electron-dominant charge transport in the CBP:(fluoranil)_2_ cocrystal is governed by the “superexchange” hopping mechanism along the D–A mixed π-stack and is dominated by factors like the energy and symmetry of the frontier molecular orbitals of the CBP and fluoranil moieties. The narrow bandgap (≈1.2 eV) and the high value of the superexchange electron transfer integral (≈100 meV) confirm the potential application of this cocrystal as the active layer material in n-type organic field effect transistors (OFETs). In addition, the strong absorption spanning from the UV to near-infrared region, narrow and direct bandgap, and low exciton binding energy indicate that the CBP:(fluoranil)_2_ cocrystal can also be exploited for photovoltaic applications. The electron–hole distribution offset, exciton size, and one-electron transition density matrix analyses confirm facile charge transfer exciton generation and dissociation leading to free charge carriers. The calculated value of spectroscopy-limited maximum efficiency (SLME) from periodic density functional theory (DFT) calculations for this cocrystal shows that it can reach a photoconversion efficiency (PCE) of 31%, implying its potential applicability as a practical photovoltaic material.

## Graphical abstract

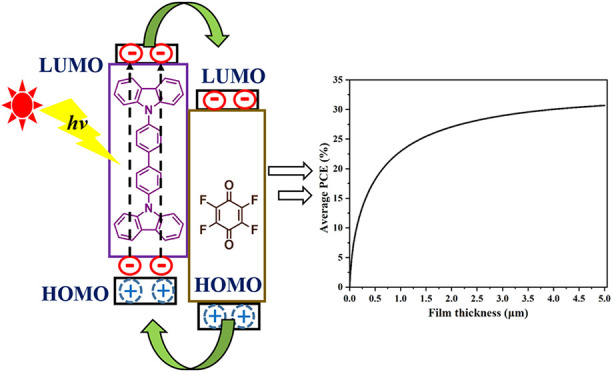

## Introduction

In the search for optoelectronic materials with mechanical flexibility, lightweight, and solution-processing ability, organic materials such as polymers^1–4^ and molecular solid aggregates^5–8^ have emerged as promising alternatives to toxic lead halide perovskites and heavy inorganic metal oxides. A number of organic polymeric and molecular crystalline materials have been extensively studied for their superior semiconducting and photophysical properties. The π-conjugated organic polymer poly­(3-hexylthiophene) (P3HT),^9–11^ an ionic aggregate of the π-conjugated organic polymer poly­(3,4-ethylenedioxythiophene):poly­(styrenesulfonic acid) (PEDOT:PSS),^12,13^ as well as crystalline molecular solids, *viz*., pentacene,^14,15^ rubrene,^16,17^ and fullerene^18,19^ have been successfully tested commercially for applications in organic light-emitting diodes (OLEDs),^20,21^ organic photovoltaics (OPVs),^22,23^ and organic field effect transistors (OFETs).^24–26^ The major drawbacks associated with the organic optoelectronic materials are hole-dominant transport and strong absorption mostly in the lower wavelength of the visible region, thus not utilizing the wide range of the solar spectrum effectively for the exciton generation, *i.e*., bound electron–hole pair generation. Therefore, significant effort has been made to develop organic molecular semiconductors with high electron mobility values^27–29^ and strong absorption in the infrared region.^30–32^

In recent years, charge transfer from an electron-rich π-donor to an electron-deficient π-acceptor has been used as an effective path to achieve both electron-dominant transport^33–35^ and strong optical absorption in the infrared region.^36–39^ Doping of π-electron acceptors in π-conjugated p-type polymer semiconductors has been proven as an effective tool to procure n-type semiconductor materials with high electron mobility values.^33–35^ Similarly, doping of a π-electron acceptor molecule in the crystal lattice of a π-donor molecule in the form of cocrystallization has emerged as a method to achieve both the ambipolar and n-type semiconductor properties,^33–35^ and strong optical absorption in the near-infrared region.^36–39^ Numerous organic cocrystals comprising π-donor and π-acceptor molecules and tethered by face-to-face π··π stacking of the donor and acceptor molecules have been successfully tested for ambipolar or electron-dominant transport, with the highest electron mobility value reaching 1.5 cm^2^ V^–1^ s^–1^, paving their way as the active layer material in next-generation ambipolar or *n*-type organic field effect transistors.^40^ In addition, strong charge transfer from the donor to the acceptor leads to strong absorption in the near- and far-infrared region,^40–44^ and such donor–acceptor cocrystals have been recently tested for photovoltaic,^41^ photothermal,^42^ solid-state lasing,^43^ and infrared optical wave-guiding^44^ applications.

The crucial factors to achieve strong charge transfer in organic cocrystals are a small energy difference, typically ≤1.5 eV, between the HOMO of the donor molecule and the LUMO of the acceptor molecule,^45^ and suitable geometrical alignment of the donor and acceptor moieties in the crystal packing to facilitate charge transfer.^46^ The carbazole moiety with a highly electron-rich π-aromatic core and a HOMO with a high energy level (−5.47 eV) is an appropriate π-donor for cocrystallization. Several donor–acceptor cocrystals with carbazole-based donors have high electron mobility values suitable for OFET applications,^47^ and strong absorption in the red or near-infrared region suitable for solid-state lasing^48,49^ and photothermal properties.^50^ Our present study elucidates the semiconducting and photophysical properties of a 1:2 π-stacked charge transfer cocrystal comprising bis-carbazole π-donor 4,4′-bis­(carbazol-9-yl)­biphenyl (CBP) and π-acceptor 1,4-tetrafluoro-*p*-benzoquinone (fluoranil), as shown in Scheme 1, to determine its potential for application in organic photovoltaic (OPV) and organic field-effect transistor (OFET) devices.

**Scheme 1:**
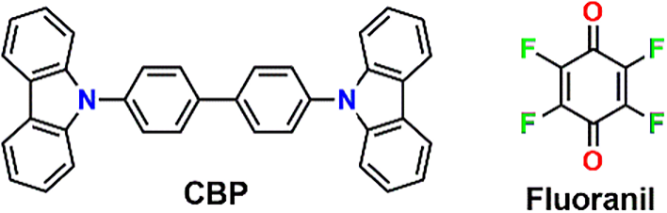
Coformers in CBP:(fluoranil)_2_ Cocrystals

The bis-carbazole π-donor has been reportedly used for charge transfer cocrystallization to procure n-type semiconductors with low bandgaps^51^ and solid-state lasers.^52^ The bis-carbazole donor CBP has a highly electron-rich carbazole moiety (Figure 1b), and a high HOMO energy level (−5.29 eV) suitable for a viable π-donor (Figure 1a). On the other hand, the π-acceptor fluoranil possesses a LUMO with an elevated energy level (−4.22 eV) (Figure 1a) and a deeply electron-depleted quinonoid core (Figure 1b). The strong π-acceptor property of fluoranil has been reportedly exploited for achieving deep red emission,^53,54^ and high electron mobility values^55,56^ in donor–acceptor (D–A) cocrystals. The complementarity of the electrostatic potential and the appropriate energy difference between the HOMO of CBP and the LUMO of fluoranil are conducive to strong charge transfer from CBP to fluoranil in the excited states and also favor the “superexchange” pathway-mediated electron transfer. This is due to the fact that the “superexchange” electron transfer mechanism, *i.e*., the transfer of electrons between the LUMO of two consecutive acceptor molecules via the HOMO of an adjoining donor molecule along a mixed π-stack, will only be energetically favorable if the energy difference between the donor HOMO and acceptor LUMO is small. Hence, the cocrystallization of CBP and fluoranil is expected to result in efficient excited-state charge transfer and favorable electron transport along the mixed π-stack.

**Figure 1:**
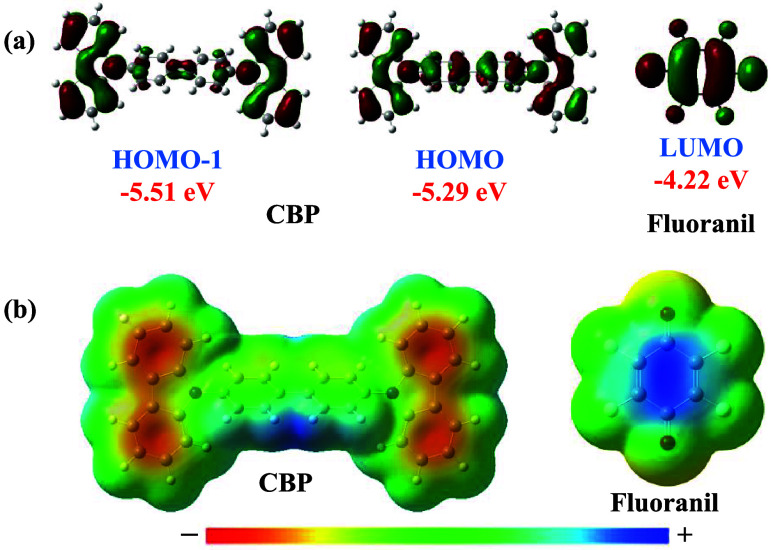
(a) Energies of HOMO/HOMO–1 of CBP and the LUMO of fluoranil calculated at the B3LYP/6-31G­(d,p) level using optimized gas-phase geometry; (b) molecular electrostatic potential of CBP and fluoranil calculated at the B3LYP/6-31G­(d,p) level using optimized gas-phase geometry.

One unique feature associated with CBP as a π-donor is that the molecular conformation controls the reorganization energy (λ) required for the change in molecular geometry upon electron ejection or acceptance, as it is involved in the change in dihedral angles between the carbazole/phenyl (θ) and phenyl/phenyl (φ) moieties. Similarly, the carbazole/phenyl and phenyl/phenyl dihedral angles (Figure 2a) observed in the cocrystals of CBP reportedly have a decisive influence on both the charge carrier polarity^51^ and emission properties.^52^ In the present study, we will demonstrate how the crystal packing features of the CBP:(fluoranil)_2_ cocrystal, as well as the inherent electronic properties of the coformers CBP and fluoranil, dictate the charge carrier transport and excited-state photophysical properties toward potential photovoltaic behavior.

**Figure 2:**
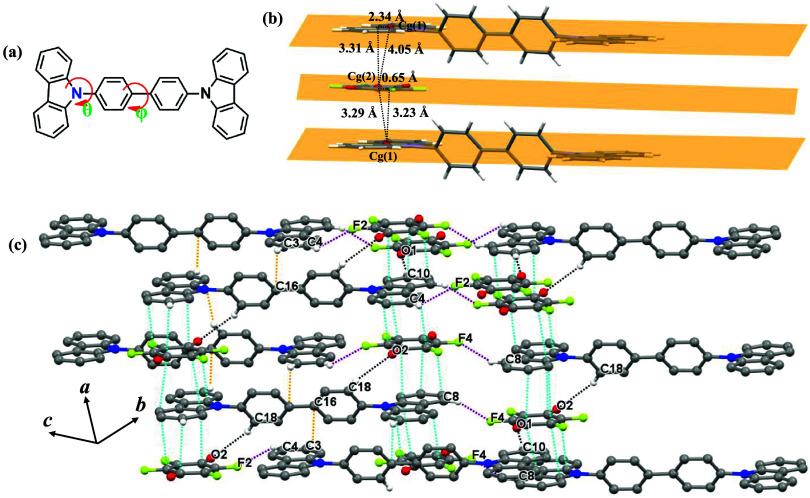
(a) Dihedral angles θ and φ in CBP molecules; (b) face-to-face π···π stacking of CBP and fluoranil molecules. The slip distances, centroid–centroid distances, and perpendicular distances of Cg(1) and Cg(2) on the adjacent ring plane are given in Å. The pairs of slip distances, perpendicular distances, and angles should be similar for perfectly parallel planes. The small differences seen here are the result of a 0.90° angle between the ring planes; (c) crystal packing of the CBP:(fluoranil)_2_ cocrystal dominated by intermolecular π···π stacking (sky blue), and C–H···O (black), C–H···F (purple), and C–H···π (orange) hydrogen-bonding interactions; aromatic hydrogen atoms not taking part in intermolecular interactions are omitted for clarity.

## Materials and Methods

### Materials

4,4′-Bis­(carbazol-9-yl)­biphenyl (99% purity), tetrafluoro-1,4-*p*-benzoquinone (97% purity), and toluene solvent were purchased from Sigma-Aldrich and used as received without further chemical purification.

### Synthesis and Structural Characterization of CBP:(fluoranil)_2_ Cocrystals

The CBP:(fluoranil)_2_ cocrystal was synthesized by grinding CBP (49 mg, 1 mmol) and fluoranil (36 mg, 2 mmol) together with repetitive addition of 2 mL of toluene at 5 min intervals. The resultant black powder (Figure S1 in SI) was dissolved in 10 mL of toluene and kept undisturbed for slow evaporation. Long needle-shaped black crystals were grown after a week and used for single-crystal X-ray diffraction studies.

Single-crystal X-ray diffraction data were collected using Mo-Kα radiation (wavelength 0.71073 Å) at a temperature of 100 K. The crystal structure was solved using direct methods with SHEXL software. The non-hydrogen atoms were refined anisotropically with full matrix least squares on *F* ^2^, while the hydrogen atoms were positioned at an idealized geometry with a fixed C_sp_^2^–H bond length of 0.93 Å. The *U*_iso_(*H*) values were set at 1.2*U*_eq_ of the atoms carrying hydrogen atoms. The crystallographic and refinement parameters for the CBP:(fluoranil)_2_ cocrystal are summarized in Table S1 (in SI). An *ORTEP* diagram of the asymmetric unit of the cocrystal with a 40% probability of thermal ellipsoids is shown in Figure S2 (in SI). The crystal structure of the CBP:(fluoranil)_2_ cocrystal has been deposited in the Cambridge Structural Database with CCDC number 2481510.

### Spectroscopic Methods

Reflectance measurements were performed using an integrating sphere setup with a BENTHAM PVE 300 spectrophotometer operated using BenWin+ software. The system was configured in the 497 AC mode to acquire spectra across the 300–1750 nm wavelength range.

#### Computational Methods

##### Theoretical Estimation of Intermolecular Interaction Energies

The binding energy between the donor and acceptor in the gas phase was calculated at the crystal structure geometry using the meta hybrid-GGA functional M06-2X, with a 6-31G­(d,p) basis set employed for binding energy calculation. The counterpoise method was used to correct the basis set superposition error (BSSE), as a relatively small basis set was used for calculating the intermolecular interaction energy values.^57^ The M06-2X functional with 54% Hartree–Fock exchange is ideal to account for the dispersive nature of π···π stacking and weak hydrogen-bonding interactions, *viz*., C–H···O/C–H···F/C–H···π.^58–60^ The energy components of π···π stacking and hydrogen-bonding interactions in the solid state were calculated at the B3LYP/6-31G­(d,p) level of theory using CrystalExplorer to understand the nature of these interactions.^61,62^

##### Theoretical Prediction of Optoelectronic Properties at the Molecular Level

The Gaussian 16 program package was used for all calculations at the atomic level. The HOMO/LUMO energies and molecular electrostatic potential surfaces of the donor and acceptor molecules were calculated at the B3LYP/6-31G­(d,p) level of theory. The HOMO/LUMO energies of the D–A dimer, A–D–A–D tetramer, and A–D–A–D–A–D hexamer were calculated with Grimme’s dispersion (D3)^63,64^ -corrected B3LYP functional and 6-31G­(d,p) basis set, using the atomic coordinates extracted from the low-temperature crystal geometry. The donor-to-acceptor charge transfer was quantified from the natural bond orbital (NBO)^65^ analysis of the π-stacked D–A dimer performed at the M06–2*X*/6–31G­(d,p) level of theory. The second-order perturbation (*E* ^2^) energy^66^ values for electron transfer from the filled π-orbitals on the donor to the empty π*-orbitals on the acceptor, along with the NBO atomic charges on the donor and acceptor moieties, give an estimation of charge transfer from CBP to the fluoranil molecule.^67^

To calculate the excited-state properties, time-dependent density functional theory (TD-DFT) calculations on the D–A dimer and A–D–A–D tetramer were performed using crystal coordinates. The CAM-B3LYP functional^68,69^ was used for the TD-DFT calculation, and only spin-allowed singlet–singlet transitions were considered in the optically excited-state calculation. The range-separated exchange-correlation functional CAM-B3LYP with 19% Hartree–Fock exchange at short range, 65% Hartree–Fock exchange at long range (>10 Å), and 33% HF exchange in the intermediate range, takes into account the long-range electron–electron coupling effect in π-stacked D–A systems to produce reliable transition energy values.^70^ The electron–hole distribution, coefficient of electron–hole mixing,^71^ charge transfer length,^72^ and one-electron transition density matrix^73^ for the excited states were obtained from the TD-DFT calculation results. The Multiwfn software^71–73^ was used for plotting the excited-state electron–hole distribution maps and heat maps for the one-electron transition density matrix, as well as calculating the charge transfer length and electron–hole mixing coefficient. Another range-separated hybrid functional ω97X-D was used to calculate the excited-state properties of the π-stacked D–A dimer, and the values were compared with those obtained by the CAM-B3LYP functional. The ωB97X-D functional comprises 22% Hartree–Fock exchange at short range and 100% Hartree–Fock exchange at long range, while the intermediate region is defined with a standard error function with a range separation parameter value of 0.2. In addition, the density of states (DOS) in the π-stacked D–A dimer of CBP:(fluoranil)_2_ was calculated using the CAM-B3LYP functional and Multiwfn software.

##### Modeling of Charge Carrier Transfer

The nonadiabatic internal hole/electron reorganization energy (λ_int_) of the molecules was calculated at the B3LYP/6-31G­(d,p) level by summing the molecular reorganization energy at both the ground (λ_*i*_) and excited (λ_f_) states upon cation/anion formation. A four-point energy model^74^ was followed to estimate the value of internal reorganization energy (λ_int_) of the molecules

1 $$\begin{array}{l} \lambda =\lambda_{i}+\lambda_{f}=\left(E_{\mathrm{cation/anion}}^{**}-E_{\mathrm{neutral}}\right)+\left(E_{\mathrm{cation/anion}}^{*}-E_{\mathrm{cation/anion}}\right)\\ \lambda_{i}=\left(E_{\mathrm{cation/anion}}^{**}-E_{\mathrm{neutral}}\right),\mathrm{and}\;\lambda_{f}=\left(E_{\mathrm{cation/anion}}^{*}-E_{\mathrm{cation/anion}}\right) \end{array}$$

Here, *E*_neutral_ and E**_cation/anion_ refer to the energy of the optimized geometry of the neutral molecule and the single-point energy of the neutral molecule with the optimized geometry of cationic/anionic states, respectively. Similarly, *E*_cation/anion_ and E*_cation/anion_ refer to the energies of the optimized geometry of the cation/anion and the single-point energy of the cation/anion with the optimized geometry of the neutral state of the molecule, respectively.

The electron and hole transfer integrals were calculated at the CAM-B3LYP/6-31G­(d,p) level of theory. The long-range corrected CAM-B3LYP functional is ideal for the transfer integral calculation as the frontier molecular orbitals in the π-stacked molecular dimers (D–D/A–A)/trimers (A–D–A/D–A–D) are generally distributed on two different molecules, necessitating the long-range electron correlation correction for the energy calculation of molecular orbitals.

##### Band Structure Calculation

The band structure calculation for the CBP:(fluoranil)_2_ cocrystal was performed at a low temperature (100 K), using the crystal geometry reported in this work, with the Vienna *ab initio* simulation package (VASP, version 6.4.3). The band structure calculation of the crystal geometry was performed with an energy cutoff of 500 eV and a Γ-centered 3 × 3 × 1 mesh of *k*-points for the SCF total energy calculation, and a density of 10 *k*-points between the high symmetry points to draw the band structure. A convergence criterion of 10^–7^ eV/Å was applied to calculate the band structure using the hybrid functional HSE06, which has been recognized to give accurate values of the band structure for organic systems.^75,76^ A Gaussian smearing scheme with a smearing width of 0.05 eV was used for the band structure calculation.

The software VASPKIT (version 1.3.5) was used for postprocessing of all data, including the calculation of the spectroscopy-limited maximum efficiency (SLME) parameter. The SLME parameter (η) is defined as the maximum photoconversion efficiency of a material. The η value equals *P*_m_/*P*_in_, where *P*_m_ is the maximum power density obtained from the material, and *P*_in_ is the incident power density from the solar spectrum. The SLME parameter is extracted by using an exponential function to model the fraction of radiative recombination as follows:

2 $$J_{\mathrm{SC}}=J_{0}\exp\int_{0}^{\infty }a\left(E\right)I_{\mathrm{sun}}EdE$$

where *J*_SC_ is the photovoltaic current, *a*(*E*) is the photon absorptivity, *I*_sun_ is the AM 1.5 G solar spectrum, *J*_0_ is the reverse photocurrent, which depends on the total electron–hole recombination, *i.e*., both the radiative and nonradiative recombination in the dark. The expression

3 $$J_{0}={J_{0}}^{r}+{J_{0}}^{\mathrm{nr}}={J_{0}}^{r}/\exp\left(E_{g}-{E_{g}}^{\mathrm{da}}/\mathrm{kT}\right)$$

where *E*_g_ is the fundamental bandgap, and *E*_g_^da^ is the direct allowed bandgap. As a result, the value of the SLME parameter becomes zero as the difference between the direct allowed and the fundamental bandgap increases. However, the SLME parameter provides a reasonable idea of the efficiency of direct bandgap photovoltaics, like the CBP:(fluoranil)_2_ cocrystal of our current study. In this regard, the spectroscopic-limited maximum efficiency (SLME) parameter provides a more practical estimation of the accurate efficiency compared to the traditional Shokley–Queisser limit, which only takes into account the bandgap.^77^

## Results and Discussion

### Supramolecular Features of the CBP:(fluoranil)_2_ Cocrystal

The 1:2 CBP:(fluoranil)_2_ cocrystal crystallizes in the centrosymmetric, triclinic space group *P*1̅. The asymmetric unit consists of one molecule of CBP and one molecule of fluoranil (Figure S3 in SI), and the unit cell comprises three such asymmetric units. The biphenyl moiety of the CBP molecule is planar, with a dihedral angle (φ) between the two phenyl rings of 0.34°. On the other hand, the dihedral angle (θ) values between the carbazole and phenyl moieties (58.29° and 54.55°) in CBP indicate that these rings are not coplanar (Figure 2a). The carbazole ring of the CBP molecule and the fluoranil molecule are tethered by *face-to-face* π–π stacking interactions along the crystallographic *a-*axis (Figure 2c). The distances between the centroids (*C*_g_) of the carbazole ring of CBP and the quinonoid ring of the fluoranil molecule are 4.05 and 3.29 Å, respectively, while the slip distances between the two rings are 2.39 and 0.65 Å, respectively. The angle between the π-molecular planes of the carbazole ring of the CBP molecule and the quinoid ring of fluoranil is 0.90°, indicating that these rings are almost coplanar (Figure 2b). These parameters indicate a moderately strong *face-to-face* π···π stacking interaction between the carbazole unit of CBP and the fluoranil molecule. The adjacent π-stacks of CBP and fluoranil molecules are joined by the C8–H8···F4 (C···F, 3.146(2) Å and C–H···F, 119.2°), C4–H4···F2 (C···F, 3.429(2) Å and C–H···F, 135.9°), and C18–H18···O2 (C···O, 3.415(2) Å and C–H···O, 134.2°) hydrogen bonds along the crystallographic *b-*axis (Figure 2c and Table 1). In addition, the CBP and fluoranil molecules form a C10–H10···O1 (C···O, 3.413(2) Å and C–H···O, 164.1°) hydrogen bond along the crystallographic *a-*axis (Table 1). The CBP molecules are joined to each other via weak C–H···π hydrogen bonds (C···C, 3.513(2) Å, and C–H···C, 133.7°) and thus form a robust 3D supramolecular network (Figure 2c).

**Table 1:** Parameters of Hydrogen-Bonding Interactions in CBP:(fluoranil)_2_ Cocrystals

interaction	D···A (in Å)	H···A (in Å)	D–H···A (in°)	symmetry
C4–H4···F2	3.429(2)	2.68	135.9	1 – *x*, −*y*, 1 – *z*
C10–H10···O1	3.413(2)	2.49	164.1	*x* – 1, *y* + 1, *z*
C18–H18···O2	3.415(2)	2.76	134.2	–*x*, 1 – *y*, 1 – *z*
C8–H8···F4	3.146(2)	2.57	119.2	*x*, *y*, *z*

The optoelectronic properties of organic molecular crystals, including charge carrier transport, are monitored by the geometrical overlap between the molecules and the strength of intermolecular interactions in crystal packing. Hence, it is necessary to estimate the strength of the intermolecular interactions in crystal packing. The intermolecular interaction energies were calculated from gas-phase molecular dimers using the counterpoise method with experimentally obtained atomic coordinates. The gas-phase binding energy for the π···π-stacked D–A dimer is −12.05 kcal/mol, while the binding energy for the C8–H8···F4 hydrogen-bonded motif is −1.62 kcal/mol. On the other hand, the gas-phase binding energies for C4–H4···F2 and C10–H10···O1 hydrogen-bonded dimers are −1.42 and −1.66 kcal/mol, respectively. The low values of these hydrogen-bonding interactions indicate that they are not as effective as the face-to-face π···π stacking interactions between CBP and fluoranil to dictate the optoelectronic properties of the CBP:(fluoranil)_2_ cocrystal. The C–H···π hydrogen bonds between the phenyl and carbazole rings of the two CBP molecules are characterized by a high value of gas-phase binding energy (−13.51 kcal/mol), reflecting substantial geometrical overlap between the molecules. This also indicates that the C–H···π hydrogen bonding between the CBP molecules has a prominent effect on charge carrier transport. We calculated the binding energy values and the nature of the intermolecular interactions for the π-stacked CBP and fluoranil molecules and the C–H···π hydrogen-bonded CBP molecules in the solid phase at the B3LYP/6-31G­(d,p) level of theory using CrystalExplorer and the crystal coordinates. The interaction energy values for π···π stacked and C–H···π hydrogen-bonded molecular dimers in the solid state are −11.62 and −11.43 kcal/mol, respectively. However, the nature of the π··π stacking and C–H···π hydrogen-bonding interactions is different, as the former is a predominantly electrostatic force, while the latter is dominated by the van der Waals attractive force (Table 2). The dominant electrostatic nature of the π···π stacking interactions will lead to significant charge transfer in both the ground and excited states.

**Table 2:** Energy Components of π···π Stacking and C–H···π Interactions in the Solid State Calculated at the B3LYP/6-31G­(d,p) Level of Theory

interaction	*E*_electrostatic_ (kcal/mol)	*E*_polarization_ (kcal/mol)	*E*_dispersion_ (kcal/mol)	*E*_repulsion_ (kcal/mol)	*E*_total_ (kcal/mol)
π···π stacking	–3.93	–1.60	–15.30	11.43	–11.62
C–H···π bond	–3.62	–1.00	–14.21	9.38	–11.43

### Ground-State Electronic Properties of the CBP:(fluoranil)_2_ Cocrystal

The analyses of the frontier molecular orbitals of the alternatively π-stacked CBP:fluoranil dimers, tetramers, and hexamers in the ground state demonstrate significant molecular orbital offsets with the HOMO/HOMO–*n* orbitals concentrated on the CBP molecule and the LUMO/LUMO+*n* (*n* = 1–3) orbitals located on the fluoranil molecule (Figures 3a and S3–S5 in SI). The HOMO/LUMO energy difference for the π-bonded dimer CBP:fluoranil, π-bonded trimer CBP:(fluoranil)_2_, π-bonded D–A–D–A tetramer, and π-bonded D–A–D–A–D–A hexamer are 1.75, 1.77, 1.43, and 1.60 eV, respectively (Figures S3–S5 in SI) with Grimme’s dispersion-corrected B3LYP-D3 functional. The small value of the HOMO/LUMO energy difference observed in the CBP:(fluoranil)_2_ cocrystal is favorable for both charge carrier transport and photovoltaic applications. In contrast, the HOMO/LUMO energy difference of the π-stacked D–A dimer calculated using the long-range electronic effect-corrected CAM-B3LYP functional is higher (3.25 eV) than that calculated using the dispersion-corrected method, but the HOMO/LUMO geometrical offset is similar to that observed in the calculation using the dispersion-corrected method (Figure S6 in SI). The plots of total density of states (TDOS) and partial density of states (PDOS) show that the contribution of HOMO is only from the donor CBP, while the LUMO originated exclusively from tthe acceptor fluoranil (Figure 3b).

**Figure 3:**
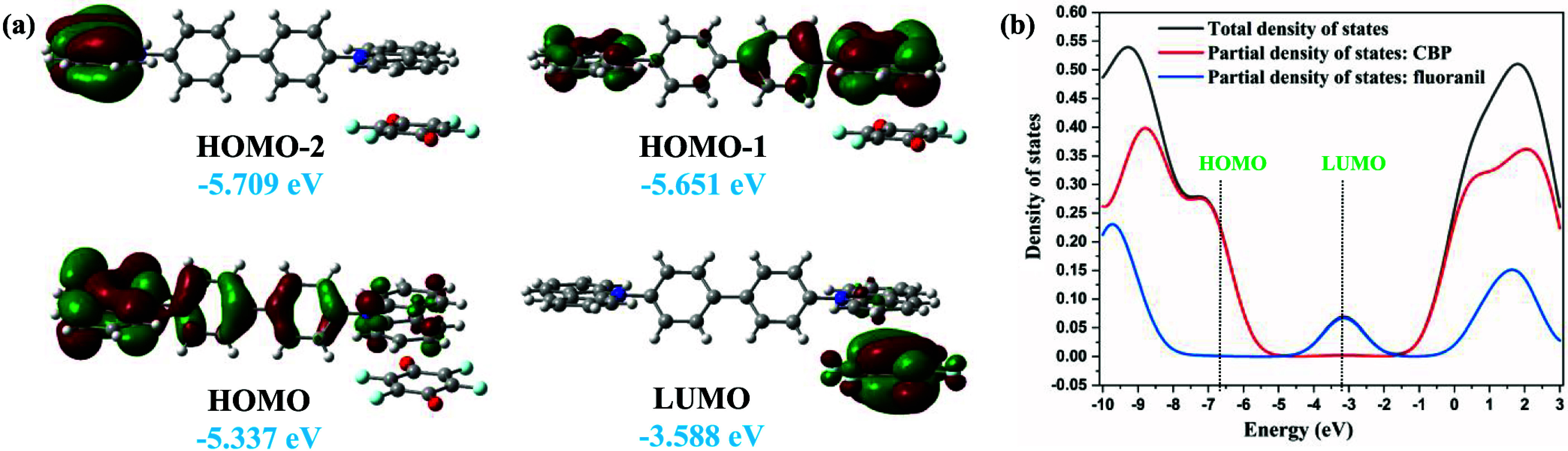
(a) Frontier molecular orbitals in the π-stacked CBP:fluoranil dimer calculated at the Grimme’s dispersion-corrected B3LYP-D3/6–31G (d,p) level. (b) Density of states analysis of the π-stacked D–A dimer, showing that the HOMO is located on CBP, and the LUMO is located on fluoranil. The calculation is carried out at the CAM-B3LYP/6-31G­(d,p) level using coordinates from the 100 K crystal structure.

We estimated the degree of ground-state charge transfer from CBP to fluoranil in the π-bonded CBP:fluoranil dimer using both the Mulliken and natural bond orbital (NBO) methods, and the degrees of charge transfer were 0.03 and 0.017, respectively, by these methods. In addition, the NBO analysis confirms electron transfer from the filled π-orbitals on the carbazole ring of CBP to the empty π*-orbitals on the quinonoid ring of fluoranil in the ground state, as evidenced by the second-order perturbation energy *E* ^2^ values shown in Figure 4.

**Figure 4:**
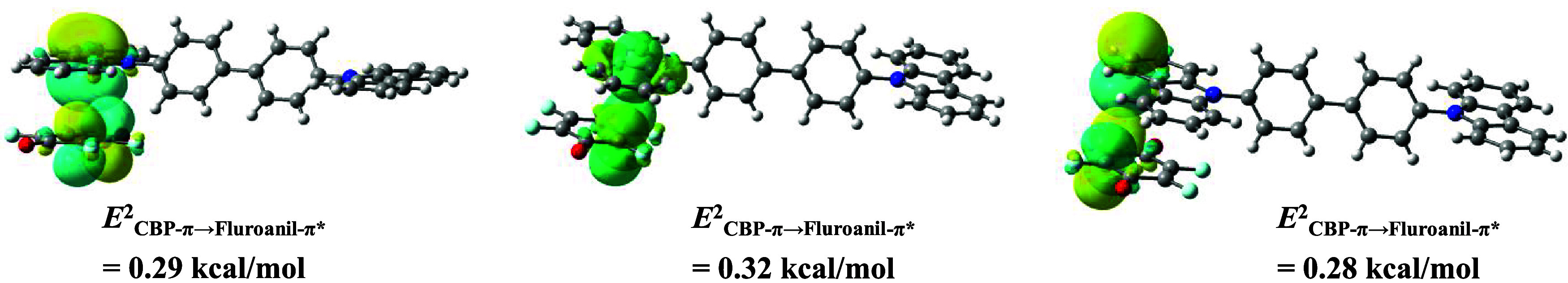
Natural bond orbital analysis confirms charge transfer from the carbazole moiety of CBP to fluoranil, and second-order perturbation energy (*E* ^2^) values confirm charge transfer.

### Spectroscopic and Excited-State Features of the CBP:(fluoranil)_2_ Cocrystal

The diffuse reflectance spectrum of the CBP:(fluoranil)_2_ cocrystal was collected using the powdered sample and converted to the solid-state absorption spectrum by using the Kubelka–Munk equation *F*(*R*) = α/*S* = (1 – *R*)^2^/(2*R*), where *F*(*R*) is the Kubelka–Munk function, α is the absorption coefficient, *S* is the scattering coefficient, which is a constant with particle size ≥ 3 μm, and *R* is the diffuse reflectance. A broad charge transfer band extending in the region of 450–800 nm is observed along the local excitation bands in the region of 325–450 nm, originating from the π→π* transitions within the CBP and fluoranil molecules (Figure 5a).

**Figure 5:**
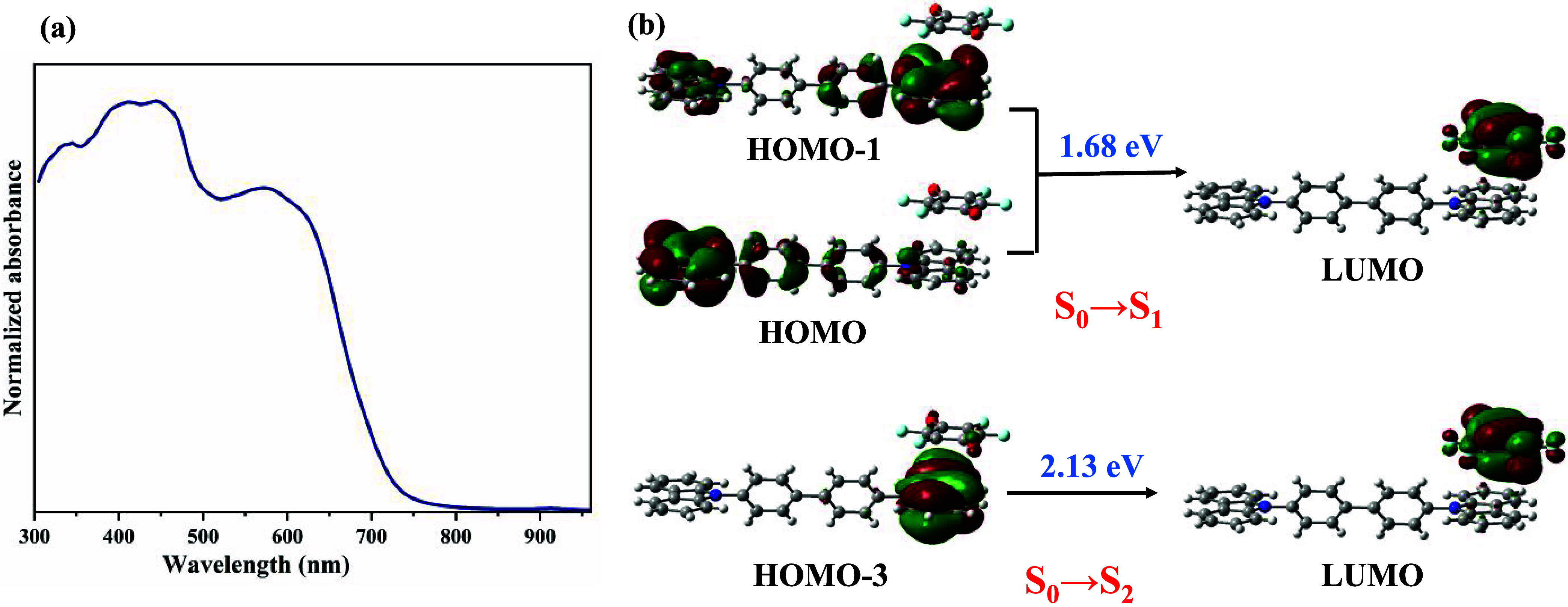
(a) Normalized solid-state absorbance spectrum of the CBP:(fluoranil)_2_ cocrystal; (b) molecular orbitals involved in the S_0_→S_1_ and S_0_→S_2_ transitions in the π-stacked CBP:fluoranil dimer.

Time-dependent DFT (TD-DFT) analyses were performed on the π-stacked D–A dimers and D–A–D–A tetramers using the coordinates obtained from low-temperature experimental geometry with the optically tuned and range-separated functional CAM-B3LYP to understand the effect of charge transfer on optical properties. The S_0_ → S_1_ (*E* = 1.68 eV) and S_0_ → S_2_ (*E* = 2.13 eV) transitions in the CBP:fluoranil dimer are associated with electron excitation from CBP to fluoranil (Figure 5b), and are characterized by moderate values of oscillator strength (Table 3). On the other hand, the S_0_ → S_3_ (*E* = 2.67 eV), S_0_ → S_4_ (*E* = 2.79 eV), and S_0_ → S_5_ (*E* = 2.85 eV) transitions are dark transitions with oscillator strength values <0.001 (Table 3). Therefore, the absorption spectrum of CBP:fluoranil is dominated by the charge transfer transition from CBP to fluoranil. The S_1_ and S_2_ states are also characterized by the high transition electric dipole moment values of 0.26 and 0.76 D, respectively, and such values of transition dipole moments are ideal for photovoltaic applications.^78,79^

**Table 3:** TD-DFT Calculated Wavelength, Oscillator Strength, and Orbital Contribution for the S_1_–S_4_ Excited States at the CAM-B3LYP/6-31G­(d, p) Level in the π-Stacked CBP:Fluoranil Dimer

state	calculated wavelength (nm)	excitation energy (eV)	oscillator strength (*f*)	orbital contribution
S_1_	738	1.68	0.0107	HOMO – 1 → LUMO, 90.11%
				HOMO → LUMO, 9.89%
S_2_	583	2.13	0.0396	HOMO – 3 → LUMO, 100%

We analyzed the electron–hole distribution, charge transfer length, and coefficient of overlap between electrons and holes for the S_1_ and S_2_ states in the π-stacked CBP:fluoranil dimer to understand the exciton generation and dissociation processes. In the first two excited states, the electrons are localized on the fluoranil molecule, while the holes are localized on the CBP molecule, implying the charge transfer nature of the excitons (Figure 6a). The charge transfer length, *i.e*., the spatial separation values of the epicenters of the electrons and holes in the S_1_ and S_2_ states, are 5.22 and 3.38 Å, respectively (Table 5), indicating a significant charge transfer character of these states, leading to the favorable dissociation of charge transfer excitons. The charge transfer length is also approximated to be the size of the exciton. In addition, the coefficients of electrons and holes mixing for the S_1_ and S_2_ states are 0.15 and 0.25, respectively (Table 5). Such low values further confirm the high likelihood of the dissociation of charge transfer excitons conducive to photovoltaic behavior. The heat maps of the one-electron transition density matrix (TDM) are valuable to understand the origin of the excited states. The prominent off-diagonal elements indicate the charge transfer origin of the states arising from excited-state electron transfer from the donor to the acceptor, while the diagonal elements indicate the local excitation character arising from the donor to donor/acceptor to acceptor transitions. The presence of bright off-diagonal elements and the absence of any prominent diagonal elements in the one-electron transition density matrix heat map of the D–A dimer demonstrate the charge transfer character of the S_0_ → S_1_ and S_0_ → S_2_ transitions, and the absence of the local excitation nature of these transitions (Figure 6b). The brightness of the off-diagonal elements in the TDM plot of the S_0_ → S_2_ transition is higher than that observed for the S_0_ → S_1_ transition, which is proportional to the oscillator strength of the transition (Table 3 and Figure 6b). We calculated the excited-state properties of the D–A dimer involving another range-separated, long-range-corrected DFT functional ω97X-D. The S_0_ → S_1_ and S_0_ → S_2_ transitions have prominent charge transfer characteristics, while the S_0_ → S_3_ transition has a local excitation nature (Figures S8–S9 and Table S2 in SI). The S_0_ → S_1_ (*E* = 2.29 eV, oscillator strength = 0.0051) and S_0_ → S_2_ (*E* = 2.71 eV, oscillator strength = 0.0534) transitions are characterized by charge transfer lengths of 6.05 and 7.49 Å and electron hole mixing coefficients of 0.23 and 0.13, respectively. In contrast, the S_0_ → S_3_ transition in the ultraviolet (UV) region (*E* = 3.32 eV, oscillator strength = 0.0024) is characterized by a charge transfer length of 5.47 Å and an electron hole mixing coefficient of 0.44, confirming the significant local excitation nature.

**Figure 6:**
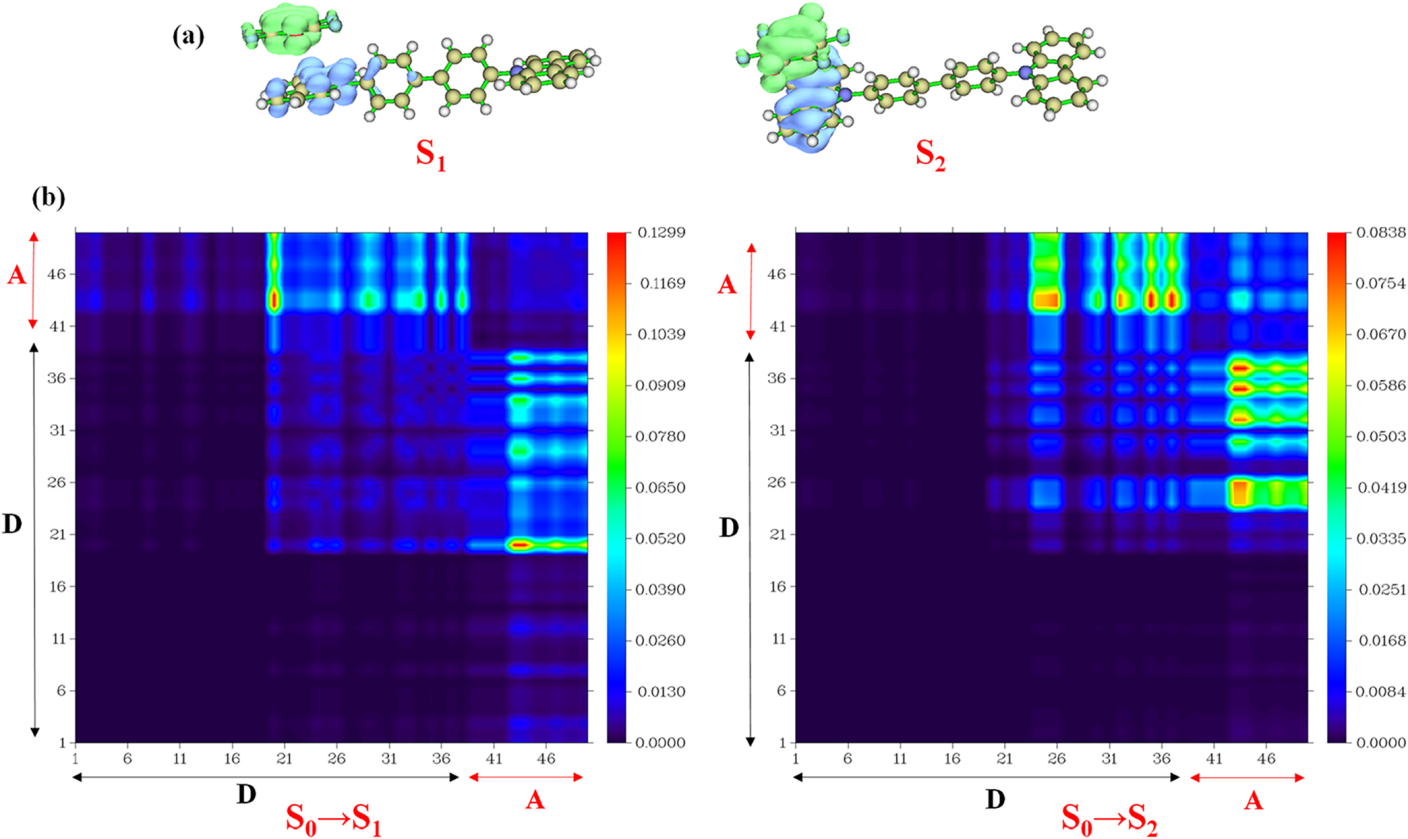
(a) Electron (green) and hole (blue) distribution in the S_1_–S_2_ excited states of the CBP:fluoranil dimer calculated at the CAM-B3LYP/6-31G­(d, p) level and plotted at an isosurface of 0.002 au; (b) one-electron transition density matrix (TDM) heat maps for the S_0_ → S_1_ and S_0_ → S_2_ transitions in the π-stacked D–A dimer; the off-diagonal elements in the heat maps indicate the charge transfer nature of the excitations.

We performed TD-DFT calculations on the π-stacked D–A–D–A tetramer to further confirm the origin of the charge transfer character of the absorption at higher wavelengths. In the tetramer, the S_1_ (*E* = 2.23 eV) and S_2_ (*E* = 2.58 eV) states with a pure charge transfer origin have moderate values of the oscillator strength (Table 4), while the S_3_ state (*E* = 2.67 eV) is a dark state with an oscillator strength value of only 0.0002. The bright S_4_ state (*E* = 2.74 eV) also has a charge transfer origin with significant oscillator strength values (Figure 7 and Table 4).

**Figure 7:**
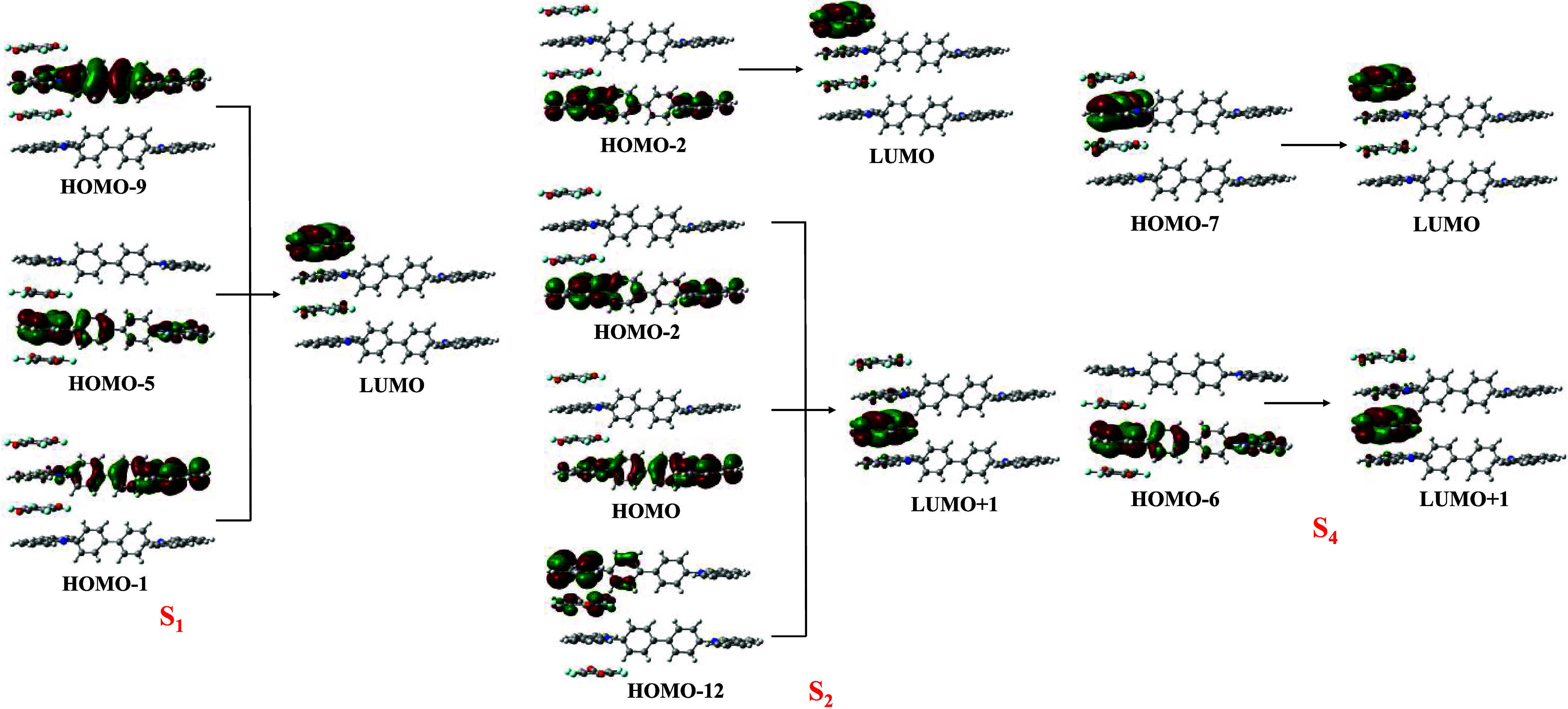
Molecular orbitals participating in the formation of the S_1_, S_2_, and S_4_ excited states in the A–D–A–D tetramer.

**Table 4:** TD-DFT Calculated Wavelength, Oscillator Strength, and Orbital Contribution for the S_1_–S_4_ Excited States at the CAM-B3LYP/6-31G­(d, p) Level in the π-Stacked D–A–D–A Tetramer

state	calculated wavelength (nm)	excitation energy (eV)	oscillator strength (*f*)	orbital contribution
S_1_	555	2.23	0.0063	HOMO – 9 → LUMO, 2.47%
				HOMO – 5 → LUMO, 84.87%
				HOMO – 1 → LUMO, 12.67%
S_2_	512	2.42	0.0077	HOMO – 12 → LUMO + 1, 2.67%
				HOMO – 2 → LUMO, 3.66%
				HOMO – 2 → LUMO + 1, 69.24%
				HOMO → LUMO + 1, 24.44%
S_4_	453	2.74	0.1037	HOMO – 7 → LUMO, 96.83%
				HOMO – 6 → LUMO + 1, 3.17%

The electron–hole analysis of the excited states in the π-stacked A–D–A–D tetramer demonstrates that the electrons are localized on the fluoranil moiety and the holes are localized on the CBP moiety in excited states S_1_, S_2_, and S_4_ (Figure 8a). The charge transfer lengths for the S_1_, S_2_, and S_4_ states in A–D–A–D tetramer are 5.40, 6.70, and 3.99 Å, respectively (Table 5), confirming the likelihood of exciton dissociation in these states. In addition, the coefficients of electron and hole mixing for the S_1_, S_2_, and S_4_ states are 0.13, 0.12, and 0.22, respectively (Table 5), and such low values of electron–hole mixing indicate a high possibility of charge transfer exciton dissociation to free charge carriers. The heat maps of the one-electron transition density matrix for the S_0_→S_1_, S_0_→S_2_, and S_0_→S_4_ transitions show prominent bright off-diagonal elements corresponding to electron excitation from the donor to the acceptor, but no significant diagonal elements due to local electron transition within the donor/acceptor moieties, thus confirming the charge transfer nature of these transitions (Figure 8b and Table 5). The off-diagonal elements are the brightest for the TDM plot of the S_0_→S_4_ transition due to the high value of the oscillator strength for this transition. The one-electron transition density matrix heat maps and electron–hole mixing coefficients indicate both facile formation and dissociation of charge transfer excitons, which are necessary criteria for photovoltaic applications. The charge transfer origin of the first few excited states can also be confirmed from the molecular orbital contribution and one-electron transition density matrix analyses of the trimeric unit comprised of one CBP molecule and two π-stacked fluoranil molecules (Figures S10–S12, and Table S3 in SI)

**Figure 8:**
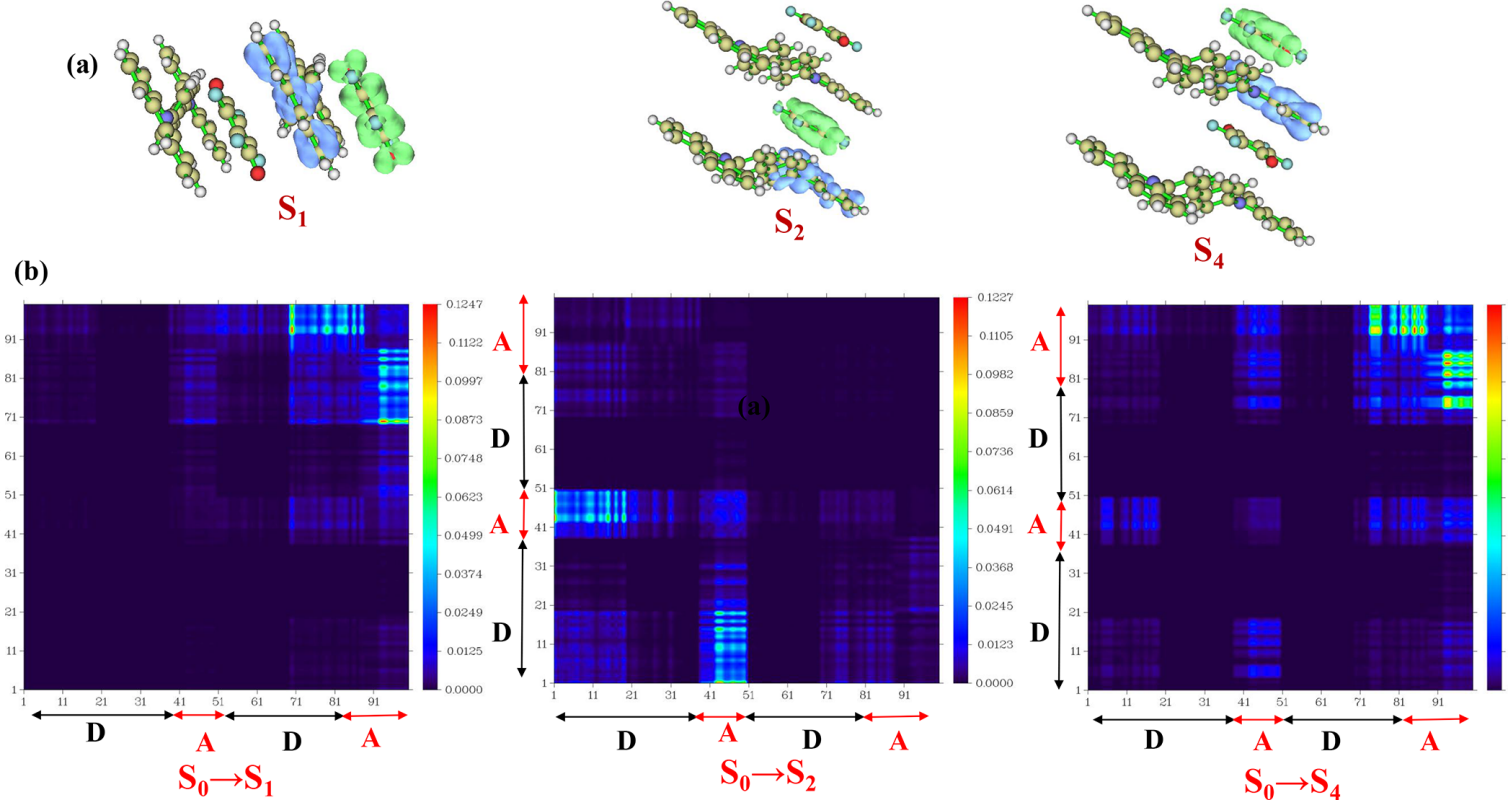
(a) Electron (green) and hole (blue) distributions in π-stacked D–A–D–A tetramer calculated at the CAM-B3LYP/6-31G­(d, p) level and drawn at an isosurface of 0.002 a.u.; (b) heat maps for the transit density matrix of S_1_, S_2_, and S_4_ states, showing prominent off-diagonal elements.

**Table 5:** Excited-State Properties Calculated at the CAM-B3LYP/6-31G­(d, p) Level in the π-Stacked D–A Dimer and D–A–D–A Tetramer to Understand Exciton Dynamics

system	excited state	charge transfer length (Å)	electron–hole mixing coefficient
D–A dimer	S_1_	5.22	0.15
	S_2_	3.38	0.25
A–D–A–D tetramer	S_1_	5.40	0.13
	S_2_	6.70	0.12
	S_4_	3.99	0.22

### Charge Carrier Transport in the CBP:(fluoranil)_2_ Cocrystal

The high mobility of free charge carriers (*i.e*., electrons and holes) is equally important as facile exciton generation and dissociation for an ideal photovoltaic material. The transport of charge carriers in organic molecular semiconductors occurs by the ‘hopping’ of charge carriers between the nearest molecules. The hopping of electrons and holes in the organic π-stacked donor–acceptor cocrystals can occur by either “superexchange” transfer along the mixed ···D–A–D–A··· stack or ‘direct’ hole transfer in donor/donor or electron transfer in acceptor/acceptor molecular dimers. In the “superexchange” mechanism, the electron “hops” between the LUMO of two consecutive acceptor molecules in a mixed ···D–A··· stack via a bridging donor HOMO/HOMO–1 orbital.^80,81^ In a similar way, the hole ‘hops’ between the HOMO of two consecutive donor molecules via the LUMO of the bridging acceptor molecule. The transfer integral, *i.e*., the probability of electron/hole hopping, depends on the geometrical overlap of the molecules, and thus, shows a direct relationship with the binding energy between the molecules. The primary charge carrier pathways in the cocrystal are the superexchange electron/hole path along the π-stack and direct hole transfer among the CBP molecules tethered by C–H··π hydrogen bonding (Figure 9).

**Figure 9:**
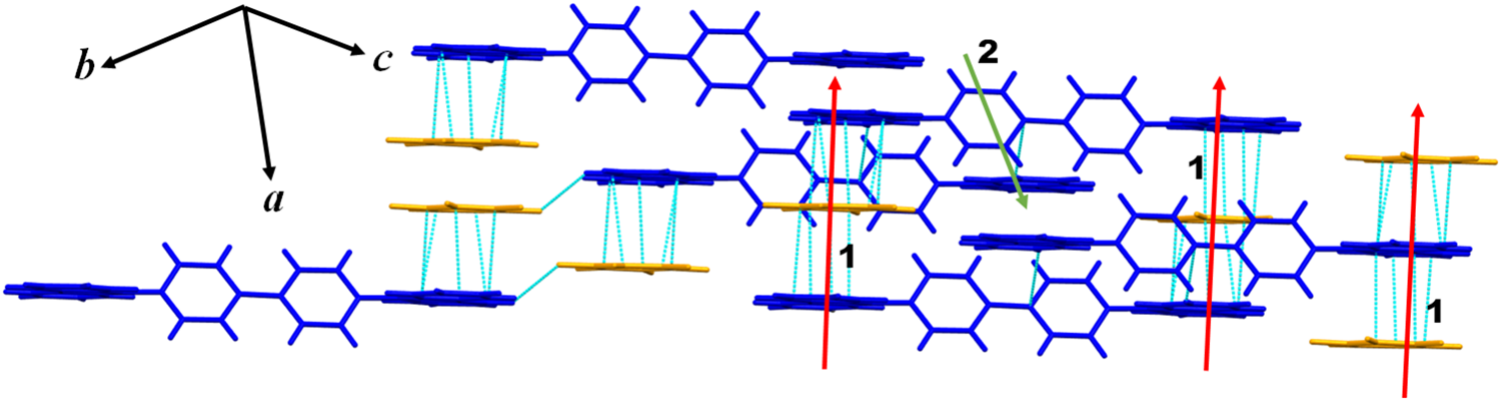
Major charge carrier transport pathways observed in the CBP:(fluoranil)_2_ cocrystal: (1) superexchange electron/hole transfer path along the mixed ···D–A–D–A··· π-stack, (2) direct hole transfer between adjacent CBP molecules adjoined by C–H··π hydrogen-bonding interactions.

The rate (*k*_T_) of electron or hole transfer via the “hopping” mechanism is expressed by the Marcus–Hush equation as follows^82,83^

4 $$\left(k_{T}\right):k_{T}=\frac{4\pi^{2}}{h}\frac{1}{\sqrt{4\pi \lambda k_{B}T}}t^{2}\exp\left[\frac{-{\left(\Delta G^{{}^{\circ}}+\lambda \right)}^{2}}{4k_{B}\lambda T}\right]$$

The decisive terms in the rate equation are the transfer integral (*t*) and the reorganization energy (λ). The transfer integral (*t*) refers to the probability of electron or hole transfer; in other words, the strength of the coupling electron (or hole) between the acceptor (or donor) molecule pairs.^81^ On the other hand, the reorganization energy (λ) indicates the energy required for the geometry change when a molecule either accepts or ejects an electron. The reorganization energy comprises internal (λ_int_) and external (λ_ext_) terms, with λ_int_ indicating the energy required for only the change in geometry of the particular molecule.^84,85^ The external reorganization energy (λ_ext_) indicates the energy required for the change in the molecular geometry of neighboring molecules, and has a much lower value compared to its internal counterpart, as the geometry change of neighboring molecules is much less prominent compared to that of the molecule accepting/ejecting an electron.^86^

The transfer integral for the ‘superexchange’ path will be calculated from the energy difference of frontier molecular orbitals of either π-stacked D–A–D or A–D–A triads following the “energy splitting method”.^87–89^ The distance between centroids of the carbazole moiety of CBP and the quinonoid moiety of fluoranil, and the dihedral angle between the planes of these moieties, are the same throughout the molecular trimers. The “superexchange” electron transfer integral is calculated from the energy difference between LUMO + 1 and LUMO of the A–D–A triad. In contrast, the “superexchange” hole transfer integral is calculated from the energy difference between the HOMO and HOMO – 1 orbital of the D–A–D triad.^87–89^ The “direct” hole transfer integral is calculated from the energy difference between the HOMO and HOMO – 1 orbitals of the D–D dimer according to the ‘site energy correction’ method.^90^ The superexchange electron and hole transfer integrals for the CBP:(fluoranil)_2_ cocrystal along the ···D–A–D–A··· stacking direction are 100.5 (Figure 10a) and 9 meV (Figure 10b), respectively (Table 6). The direct hole transfer integral for the CBP dimer is 15.0 meV, which is not very high to have balanced electron and hole transport (Figure S13 in ESI, Table 6). The superexchange and direct transfer integral values indicate electron-dominant transport in the CBP:(fluoranil)_2_ cocrystal and its potential for the application as the active channel material in *n*-type organic field-effect transistors.

**Figure 10:**
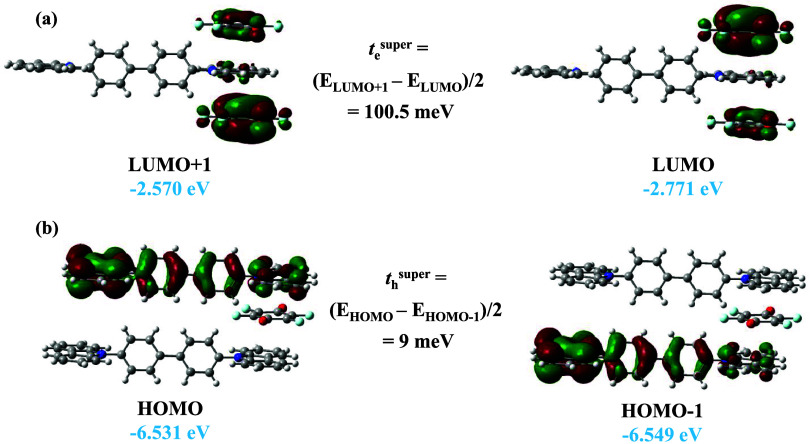
(a) Superexchange electron transfer integral calculated from the A–D–A trimer; (b) superexchange hole transfer integral calculated from the D–A–D trimer in the CBP:(fluoranil)_2_ cocrystal.

**Table 6:** Direct and Superexchange Transfer Integral Values along Different Directions Calculated from Molecular Clusters

type of transfer	molecular diads/triads for calculation	direction	transfer integral value (meV)
direct hole transfer	CBP diad	*ab*-plane	15.0
superexchange hole transfer	CBP:fluoranil π···π stacked triad	*a*-axis	9.0
superexchange electron transfer	CBP:fluoranil π···π stacked triad	*a*-axis	100.5

The superexchange transfer integrals depend on the energy and symmetry matching between the participating frontier molecular orbitals of the two molecules/moieties, which are involved in charge carrier transport and the frontier molecular orbital of the bridging molecule/moiety.^91,92^ The energy and symmetry of acceptor LUMOs participating in electron transport should match that of the bridging donor HOMO/HOMO–1 orbitals. Similarly, the energy and symmetry of donor HOMOs that participate in hole transfer must match those of the bridging acceptor LUMO. The energy and symmetry of carbazole will be considered for the superexchange process, as only the carbazole moiety of the CBP molecule acts as the π-donor, whereas the biphenyl moiety of CBP is not directly involved in the electron transfer process. The energies and symmetries of the carbazole HOMO/HOMO – 1 orbital and fluoranil LUMO/LUMO+1 orbital are shown in Figure 11. The LUMO+1 orbital of fluoranil cannot participate as a bridging orbital for the superexchange hole transfer process, as it is much higher in energy (−0.80 eV) compared to the HOMO of the CBP molecule. The HOMO (−5.47 eV) of carbazole is characterized by a “*gerade*” vertical symmetry, while the HOMO – 1 orbital (−5.72 eV) is characterized by an “*ungerade*“ vertical symmetry similar to the LUMO (−4.21 eV) of the fluoranil molecule (Figure 11). As a result, superexchange hole transport between the HOMO of consecutive carbazole moieties via the LUMO of the bridging fluoranil molecule in a π-stack is not symmetrically allowed, and the superexchange hole transfer integral is therefore negligible. In contrast, the transport of electrons between the LUMO of two fluoranil molecules can take place via either the HOMO or HOMO–1 orbital of the carbazole, considering the proximity of their energetic levels. This explains the origin of the *n*-type semiconductor nature of the CBP:(fluoranil)_2_ cocrystal.

**Figure 11:**
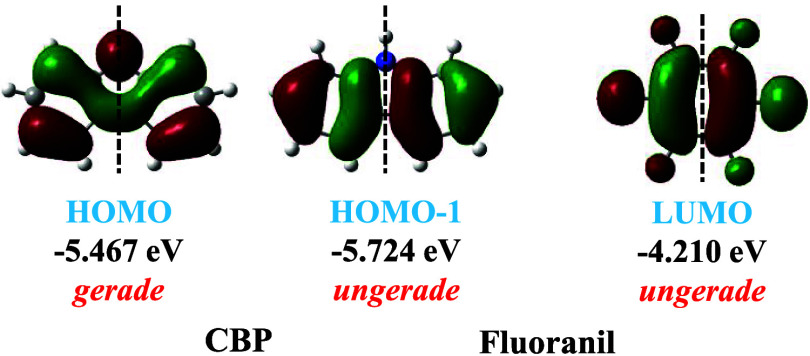
Energy and symmetry of molecular orbitals participating in the superexchange electron and hole transfer processes.

The reorganization energy of the donor and acceptor molecules has a prominent effect on determining the rate of charge carrier transport. The internal electron reorganization energy (λ_int_), *i.e*., the energy required to change the molecular geometry upon electron acceptance or anion formation for the fluoranil molecule, is 675 meV. The high value of internal electron reorganization energy of fluoranil is attributed to the considerable changes in the C–F (1.33 to 1.36 Å), C–C (1.49 to 1.45 Å), C=C (1.34 to 1.37 Å), and C=O (1.22 to 1.26 Å) bond lengths and ∠C–C–C bond angles (116.3° to 112.8°) from the optimized neutral to the optimized anion geometry. The internal hole reorganization energy of the CBP molecule, *i.e*., the energy required for the formation of the CBP^+·^ cation, is 129 meV, which is considerably lower than the electron reorganization energy of fluoranil. There are no noticeable changes in the bond lengths and phenyl–phenyl (φ) dihedral angles; however, the dihedral angle between carbazole and phenyl (θ) changes significantly from the optimized ground state to the optimized anion geometry (53.4° to 44.6°). The high value of the electron reorganization energy of fluoranil is expected to hinder the electron transport in the CBP:(fluoranil)_2_ cocrystal, despite the high value of the superexchange electron transfer integral. In contrast, the low value of the hole reorganization energy of CBP is expected to facilitate hole transfer along the C–H···π hydrogen-bonded CBP dimers, though the value of the direct transfer integral (15.0 meV) is not very high.

The values of the superexchange electron/hole transfer integral and internal reorganization values were considered to provide an idea of the relative rate of electron and hole transfer along the mixed ···D–A–D–A··· stack. We considered a barrier-free charge carrier transfer for simplification, *i.e*., Δ*G* ^‡^ = 0.^93^ As the free energy of activation Δ*G* ^‡^ = Δ*G*° + λ, the value of Δ*G*° = −λ. Under activationless or optimal reaction conditions, the reactants (*i.e*., the donor and acceptor in this case) and neighboring molecules must reorganize themselves to reach the transition state on the product side. This leads to the modification of the Marcus equation; the rate of charge carrier transfer in activationless transport is $k_{T}=\frac{4\pi^{2}}{h}\frac{1}{\sqrt{4\pi \lambda k_{B}T}}t^{2}$ , as exp(0) = 1.

In the case of activationless transport, the ratio of electron and hole transfer rate is (*t*_e_^2^/√λ_e_)/ (*t*_h_^2^/√λ_h_) = (100.5^2^/675)/[(9^2^/129)+(15^2^/129)] = 6.3, considering the superexchange electron transfer, both superexchange and direct hole transfer, and the internal electron and hole reorganization energy values. Therefore, the electron is expected to hop around 6.3 times faster compared to the hole.

We also estimated the internal and external contributions to the total reorganization energies using periodic density functional theory (VASP, functional PBEsol, energy cutoff = 700 eV, γ-centered 3 × 3 × 1 mesh, and Grimme’s D3 dispersion). The charged state is constructed by adding an electron to the conduction band, balanced by an appropriate electrostatic correction using the Jellium model of the counter charge for charged periodic systems^94^ to maintain overall charge neutrality. All basic atomic position, cell shape and cell volume are fully relaxed in both the neutral and charged states giving the E^N^_geom=N_ and E^C^_geom=C_ contributions to the total reorganization energy. The E^C^_geom=N_ and E^N^_geom=C_ are found by single point charged and neutral total energies calculations carried out in the optimized neutral and the optimized charged geometries, respectively. We then calculated λ_tot_ following the reference:^95^

2a $$\lambda^{\mathrm{tot}}=1/2\left({E^{C}}_{\mathrm{geom=N}}-{E^{C}}_{\mathrm{geom=C}}+{E^{N}}_{\mathrm{geom=C}}-{E^{N}}_{\mathrm{geom=N}}\right)=0.56\;\mathrm{eV}$$

Next, we estimated the internal contributions to λ_tot_ by the constrained optimization of the crystal structure starting from fully relaxed neutral and fully relaxed charged geometries but with the intermolecular degrees of freedom kept frozen while optimizing the intramolecular degrees of freedom only. The λ_ext_ is calculated after these constrained optimizations using an equation analogous to eq 5, giving λ_ext_ = 178 meV.

The comparison of the difference between the fully relaxed charged and neutral geometries is in good agreement with those from the molecular-level calculations. We found no notable changes in the CBP molecule; *i.e*., the differences in the C–C bond distances between the optimized charged and neutral geometries were always less than 0.005 Å. However, the distinct changes in the intermolecular distances in the fluoranil are consistent with the molecular calculations. The changes in the bond distances from neutral to charged states are C–F (1.34 to 1.35 Å), C–C (1.48 to 1.465 Å), C=C (1.35 to 1.36 Å), and C=O (1.23 to 1.25 Å), which are slightly smaller than those obtained from the relaxed molecular-level calculations: C–F (1.33 to 1.36 Å), C–C (1.49 to 1.45 Å), C=C (1.34 to 1.37 Å), and C=O (1.22 to 1.26 Å). This indicates that periodic DFT calculations are able to capture the intermolecular and intramolecular geometrical distortions following charge transfer and hence reasonably estimate the reorganization energies. The bond distances and bond angles of the fluoranil molecule in the optimized neutral and optimized charged geometries obtained from periodic DFT calculations are as follows.

Neutral: C=O = 1.231 Å, 1.232 Å; C–F = 1.338 Å, 1.342 Å, 1.343 Å, 1.343 Å; C–C = (1.48 × 3) Å, (1.35 × 2) Å; C–C–C = 115°, 122°, 115°, 122°, 115°.

Charged: C=O = 1.245 Å, 1.249 Å; C–F = 1.347 Å, 1.352 Å, 1.354 Å, 1.352 Å; C–C = (1.465 × 4) Å, (1.360 × 2) Å; C–C–C = 114.4°, 123°.

### Band Structure Calculation for the CBP:(fluoranil)_2_ Cocrystal

The band structure of CBP:(fluoranil)_2_ was calculated with the HSE06 hybrid functional at the unit cell geometry obtained experimentally at 100 K, and plotted against the high symmetry points of the first Brillouin zone (Figure 12a). The coordinates of the high symmetry points along the first Brillouin zone are listed in Table S4 (in SI). The CBP:(fluoranil)_2_ cocrystal is a direct bandgap semiconductor with both the valence band maxima and conduction band minima residing at the Γ point (0, 0, 0). The calculated bandgap of the cocrystal with the HSE06 functional is 1.24 eV. Two noticeable curvatures are found in the valence band, *viz*., along the T (0, 0.5, 0.5) → U (0.5, 0, 0.5) direction and along the X (0.5, 0, 0) → Y (0, 0.5, 0) direction, both of which indicate the crystallographic *ab* plane. This is the direction of the C–H···π hydrogen-bonded chain of the CBP molecules, *i.e*., the direction of direct hole hopping between the CBP molecules. A noticeable curvature in the conduction band was observed along the R (0.5, 0.5, 0.5) → T (0, 0.5, 0.5) direction, *i.e*., along the crystallographic *a-*axis, which is also the direction of the superexchange electron transfer (Figure 12b). It can thus be observed that the greatest curvatures in the valence and conduction bands are observed along the directions of the highest hole and electron transfer integrals, respectively. It is pertinent to mention that the hybrid exchange-correlation functional HSE06 is considered the most reliable DFT functional to produce an accurate bandgap value compared to other functionals. However, the HSE06 functional still underestimates the bandgap value by 0.2–0.3 eV.^96^

**Figure 12:**
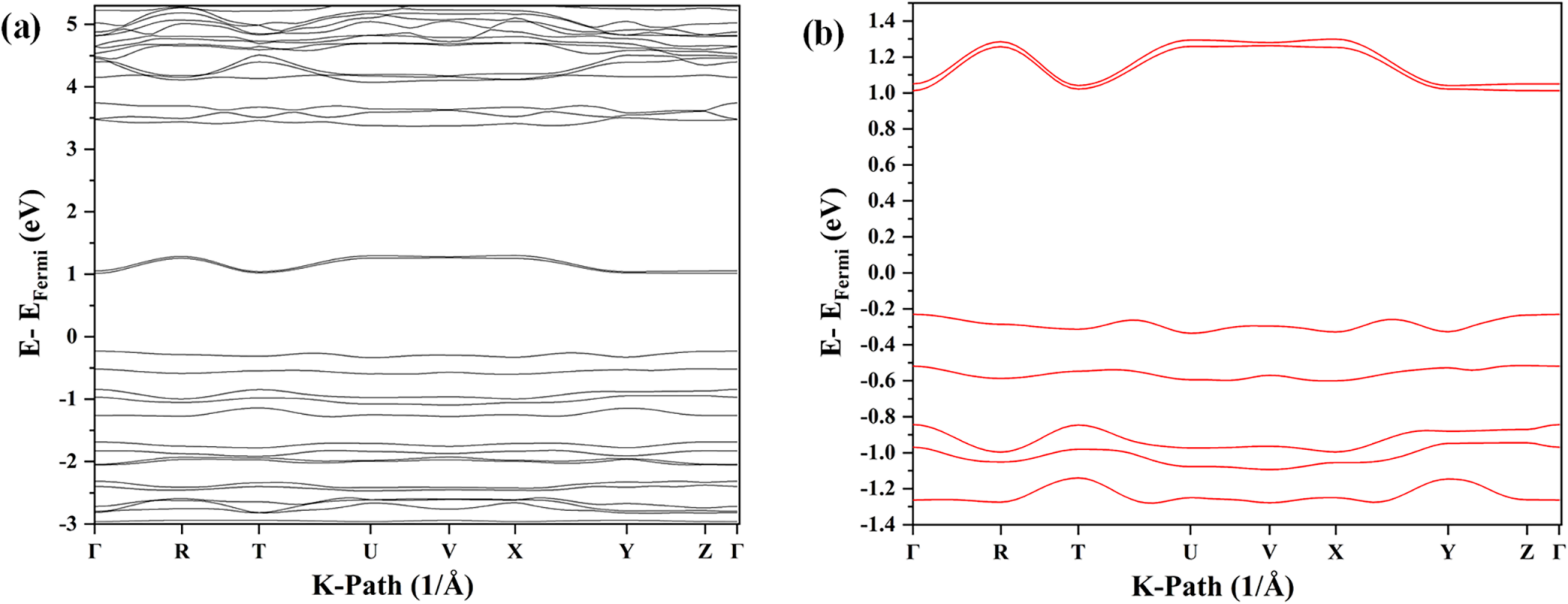
(a) Band structure of the CBP:(fluoranil)_2_ cocrystal calculated using the HSE06 functional with a low-temperature unit cell geometry with a Γ-centered 3 × 3 × 1 mesh and 500 eV cutoff; (b) the lowest two conduction and the highest two valence bands.

The exciton binding energy (*E*_b_) is approximated as the difference between the optical and electronic bandgaps. The optical bandgap of the CBP:(fluoranil)_2_ cocrystal calculated from the Tauc plot is 1.72 eV (Figure 13a), considering a direct optical bandgap according to the following equation

$${\left(\alpha h\upsilon \right)}^{2}=A\left(h\upsilon -E_{g}\right)$$

**Figure 13:**
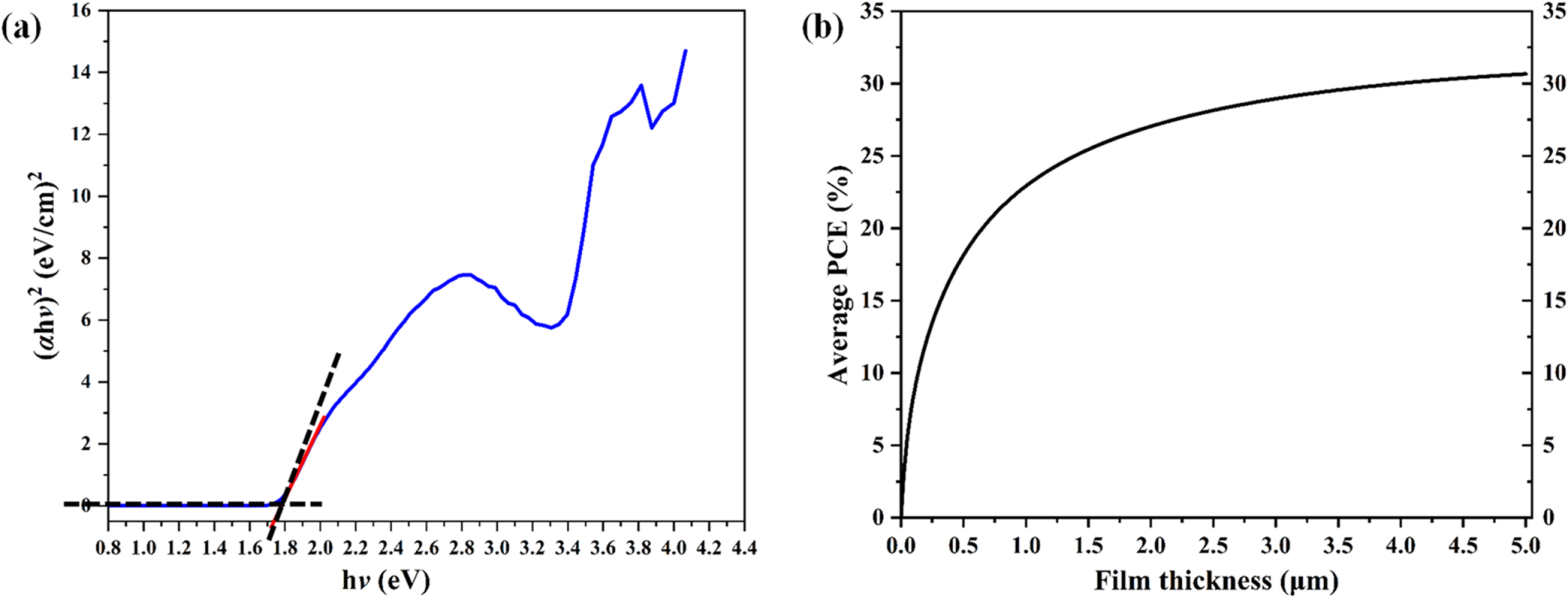
(a) Optical bandgap obtained from the Tauc plot; (b) spectroscopic-limited maximum efficiency (SLME) curve for the cocrystal shows that a 31% photoconversion efficiency can be reached with a 1.2 μm thickness.

while α = absorption coefficient, *h*υ = photon energy.

The calculated exciton binding energy, as the difference between the electronic and optical bandgaps, is around −0.5 eV. Negative values of exciton binding energy have been reported for some well-known photovoltaic materials in the solid state. The exciton binding energy in the gas phase corresponds to the electron–hole Coulomb attraction energy and is always positive. In contrast, the charge carriers in the solid state can have strong polarization effects and become more stable than neutral excitons. The exciton binding energy (*E*_b_) becomes negative when the total polarization energy of the electron and hole is stronger than the excitonic Coulomb energy. In such cases, *E*_b_ becomes more negative, and exciton dissociation to free charges is thermodynamically allowed.^97–99^ We believe that the exciton binding energy value will not be as negative as −0.5 eV, considering the known underestimation of the electronic bandgap using the HSE06 functional. However, we believe that the *E*_b_ value will still be negative, similar to that observed in the well-known organic photovoltaic material Y6 in the solid state.^100,101^ The negative exciton binding energy, along with the high values for the charge transfer length, *i.e*., the exciton size^97–99^ (Table 5), confirms a favorable exciton dissociation process suitable for photovoltaic applications. In addition, we calculated the ‘spectroscopy-limited maximum efficiency’ (SLME) parameter proposed by Lie and Zunger from a periodic DFT study to understand the efficiency of the CBP:(fluoranil)_2_ cocrystal as a potential photovoltaic material.^102^ This theoretical parameter considers the bandgap value and the nature of the bandgap (*i.e*., direct versus indirect), the optical absorption coefficient, and the nonradiative recombination losses. We calculated that the CBP:(fluoranil)_2_ cocrystal can reach 31% photoconversion efficiency, which indicates that this cocrystal can have practical applications as an organic photovoltaic (Figure 13b). In addition, DFT calculations show that it is theoretically possible to attain a 25% photoconversion efficiency (PCE) with a thin film thickness of ≈ 1200 nm of the cocrystal (Figure 13b). A recent theoretical study shows that the commercial inorganic photovoltaic material GaAs and the promising material Sb_2_Se_3_ are able to reach a PCE value of 25% with a thin film thickness of ≈500 nm.^77^ However, dense crystal packing of organic cocrystals may lead to nongeminate electron–hole recombination processes in the crystalline state of the material and can negatively affect the PCE value. On the other hand, the thin film morphology should be considered for cocrystal thin films, as it is difficult to achieve a single-crystal morphology in organic thin films. Therefore, the predicted efficiency of the CBP:(fluoranil)_2_ cocrystal as a practical photovoltaic material is not on par with that of inorganic materials, but is not insignificant for a promising organic photovoltaic material.

## Conclusion

We have described the semiconductor and excited-state photophysical properties of a π-stacked donor–acceptor cocrystal containing π-donor 4,4′-bis­(carbazol-9-yl)­biphenyl (CBP) and π-acceptor 1,4-tetrafluoro-*p*-benzoquinone (fluoranil). The CBP:(fluoranil)_2_ cocrystal is characterized by strong face-to-face π···π stacking interactions between the carbazole moiety of CBP and the fluoranil molecule, which prompts prominent charge transfer from the carbazole moiety to fluoranil. The CBP:(fluoranil)_2_ cocrystal is characterized by a direct bandgap of 1.24 eV, large exciton size (>6 Å), negative exciton binding energy, and strong absorption in the deep red and near-infrared regions, which are favorable for photovoltaic applications. DFT calculations predicted that a spectroscopy-limited maximum efficiency (SLME) value of 31% for the cocrystal, indicating the possibility of practical application as a photovoltaic material. In addition, the high value of the electron transfer integral along the mixed D–A stacking direction is favorable for application in both photovoltaic devices and n-type organic field-effect transistors. Additionally, the moderate value of the hole transfer integral along the C–H···π hydrogen-bonded CBP molecular chain and the low value of the hole reorganization of the CBP donor are advantageous for achieving moderate hole mobility. Therefore, the CBP:(fluoranil)_2_ cocrystal is expected to behave as an ambipolar semiconductor, which is ideal to facilitate photovoltaic applications. The major drawback associated with the cocrystal is the high value of the electron reorganization energy of the fluoranil acceptor, which is unfavorable for achieving a high electron mobility value. Moreover, the cocrystal LUMO energy (−3.6 eV) level is slightly higher than the ideal value (−3.8 eV) required to witness prolonged air stability. The other limitation is the significant energy differences between the HOMO levels on the donor and acceptor, as well as the LUMO levels on the donor and acceptor, which can cause electron–hole recombination. Despite these limitations, the CBP:(fluoranil)_2_ cocrystal shows promise as an *n*-type organic semiconductor with potential applications as a photovoltaic material, as evidenced by the calculated values of the bandgap and SLME parameter. Organic cocrystals have recently been recognized as an emerging class of ambipolar or *n*-type semiconductor materials with moderate mobility values; however, their photovoltaic applications have not been explored. The present study on the excited-state photophysical properties of CBP:(fluoranil)_2_ cocrystals, focusing on photovoltaic applications, is a step toward procuring a new class of molecular photovoltaics that are flexible, lightweight, and can be easily solution-processed at room temperature.

## Supplementary Material





## Notes

CBP_FL (CIF)
Crystallographic and refinement table (Table S1); formation of the cocrystal (Figure S1); *ORTEP* diagram of the cocrystal (Figure S2), molecular orbital description of the π-stacked trimer and tetramer (Figures S3–S6); time-dependent DFT (TD-DFT) calculations on the π-stacked dimers, trimers, and tetramers (Tables S2–S3 and Figures S8–S10); coordinates of high symmetry points (Table S4); calculated absorption spectra (Figures S7 and S14); electron–hole analysis and heat maps of transition density matrix for the π-stacked trimer (Figures S9 and S12); calculation of the direct hole transfer integral (Figure S13) (PDF)
