# Supplementary material for: Cocrystal Engineering of Organic Semiconductors for Photovoltaic Applications: Modeling Excited-State Properties of a Charge Transfer Cocrystal of a Dicarbazole Donor and a Fluoranil Acceptor
Source: J Phys Chem C Nanomater Interfaces. 2026 Jan 2;130(2):882–97. doi: 10.1021/acs.jpcc.5c06828 (PMC12814566; doi:10.1021/acs.jpcc.5c06828)
Supplement: Supplementary file 2 [file jp5c06828_si_002.pdf]

Cocrystal engineering towards organic semiconductor for photo-voltaic application: Modelling excited state properties of a charge transfer cocrystal of di-carbazole donor and fluoranil acceptor

Arkalekha Mandal,[a*] Chris Erik Mohn,[a*] Carl Henrik Görbitz,[a] Anurag Roy[b]

[a] Department of Chemistry, Blindern Campus, University of Oslo, Oslo 0371, Norway

[b] Environmental and Sustainability Institute, Faculty of Environment, Science and Economy, University of Exeter, Exeter, Penryn Campus, Cornwall TR10 9FE, United Kingdom

Email address: arkalekha.mandal@kjemi.uio.no

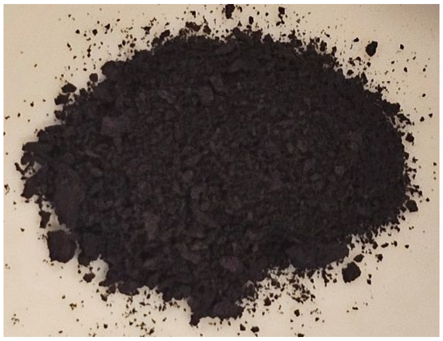

Figure S1. Black colour powder of CBP:$(fluoranil)_2$ cocrystal is shown.

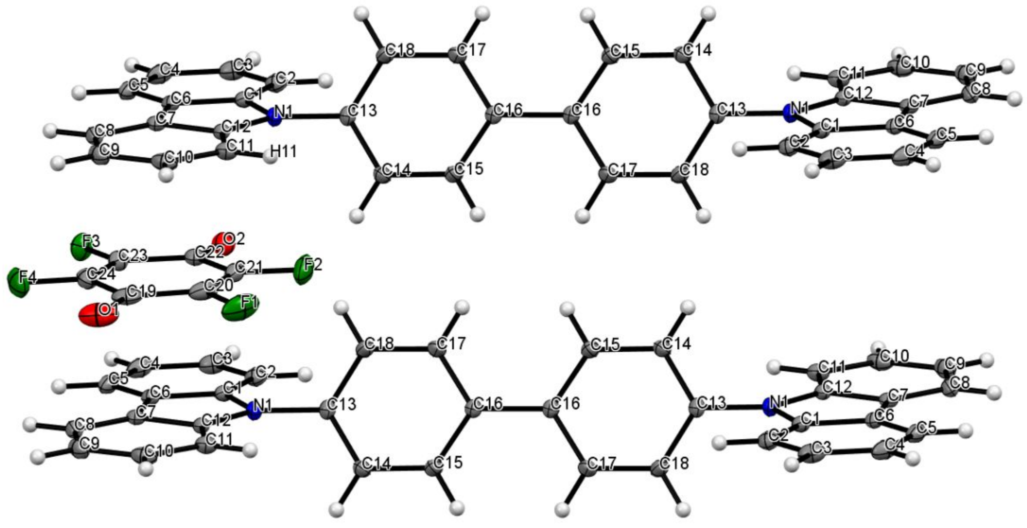

Figure S2. *ORTEP* diagram of CBP:(fluoranil)$_2$ cocrystal, thermal ellipsoids are drawn at 40% probability. The asymmetric unit is shown in the figure.

Table S1. Crystallographic and refinement parameters for CBP:(fluoranil)$_2$ cocrystal

chemical formula	$C_{48}H_{24}N_2O_4F_8$
formula weight	844.70
temp (K)	100(2)
CCDC Number	2481510
crystal system	Triclinic
space group	*P*-1
a (Å)	7.0223(4)
b (Å)	8.3105(4)
c (Å)	16.8626(8)
α (°)	76.552(1)
β (°)	78.940(1)
γ (°)	75.559(1)
V (Å^3)	917.45(8)
Z	1
F_{000}	430
ρ_{calcd} (g cm^{-3})	1.529
no. of unique reflection	4980
no. of reflection ($I \geq 2\sigma(I)$)	4078
R_1^a, R_1^b (all data, $I \geq 2\sigma(I)$)	0.0598, 0.0451
wR_2^a, wR_2^b (all data, $I \geq 2\sigma(I)$)	0.1146, 0.1078
goodness of fit (F^2)	1.061
largest peak/hole (e Å^{-3})	0.473/-0.263

Table S2. TD-DFT calculated wavelength, oscillator strength and orbital contribution for S_1-S_2 excited states at ω97X-D/6-31G(d, p) level in CBP:fluoranil dimer

**State**	**Calculated wavelength (nm)**	**Excitation energy (eV)**	**Oscillator strength (*f*)**	**Orbital contribution**
S_1	543	2.29	0.0051	HOMO-6 → LUMO, 4.28%<br>HOMO-4 → LUMO, 2.45%<br>HOMO-1 → LUMO, 73.53%<br>HOMO → LUMO, 18.94%
S_2	457	2.71	0.0534	HOMO-10 → LUMO, 3.34%<br>HOMO-3 → LUMO, 96.66%

Table S3. TD-DFT calculated wavelength, oscillator strength and orbital contribution for S_1-S_3 excited states at CAM-B3LYP/6-31G(d, p) level in CBP:(fluoranil)$_2$ trimer

**State**	**Calculated wavelength (nm)**	**Excitation energy (eV)**	**Oscillator strength (*f*)**	**Orbital contribution**
S_1	566	2.19	0.0041	HOMO-5 → LUMO, 2.53%<br>HOMO-1 → LUMO, 35.70%<br>HOMO → LUMO, 61.77%
S_2	543	2.28	0.0023	HOMO-4 → LUMO+1, 2.31%<br>HOMO-1 → LUMO+1, 3.66%<br>HOMO → LUMO+1, 24.44%
S_3	472	2.63	0.1037	HOMO-2 → LUMO, 96.83%

Table S4. Coordinates of high symmetry points in first Brillouin zone for CBP:(fluoranil)$_2$ cocrystal

High symmetry point	Coordinates
Γ	0, 0, 0
R	0.5, 0.5, 0.5
T	0, 0.5, 0.5
U	0.5, 0, 0.5
V	0.5, 0.5. 0
X	0.5, 0, 0
Y	0, 0.5, 0
Z	0, 0, 0.5
Γ	0, 0, 0

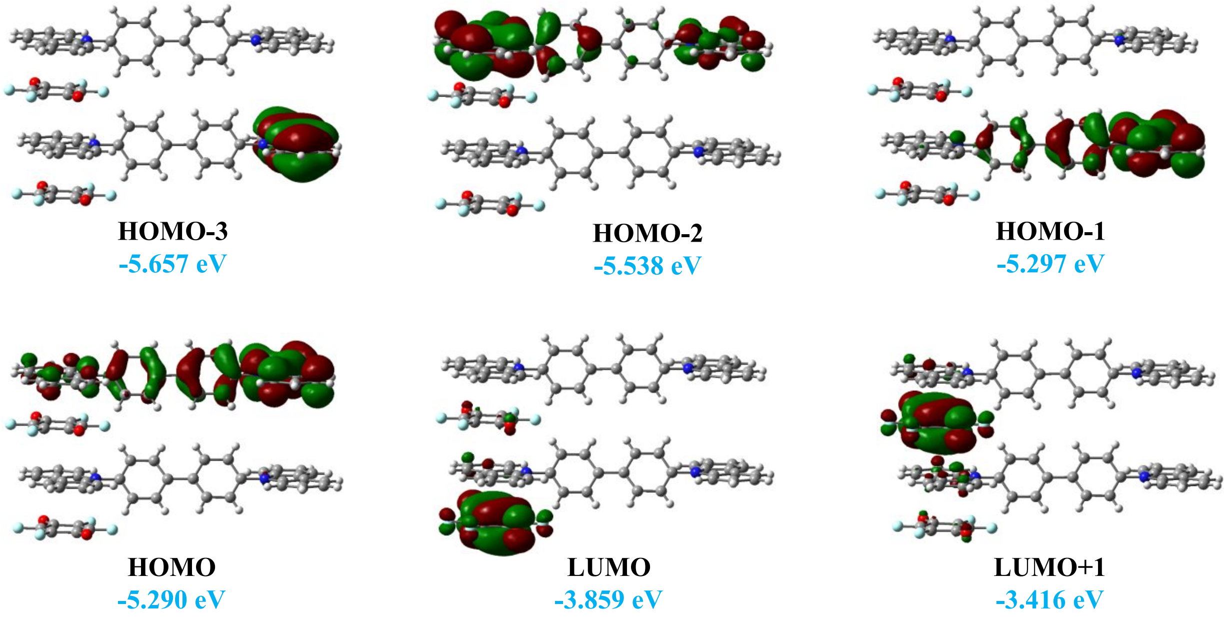

Figure S3. The frontier molecular orbital of π-stacked D–A–D–A tetramer, calculated at Grimme's dispersion corrected B3LYP-D3/6-31G(d,p) level of theory.

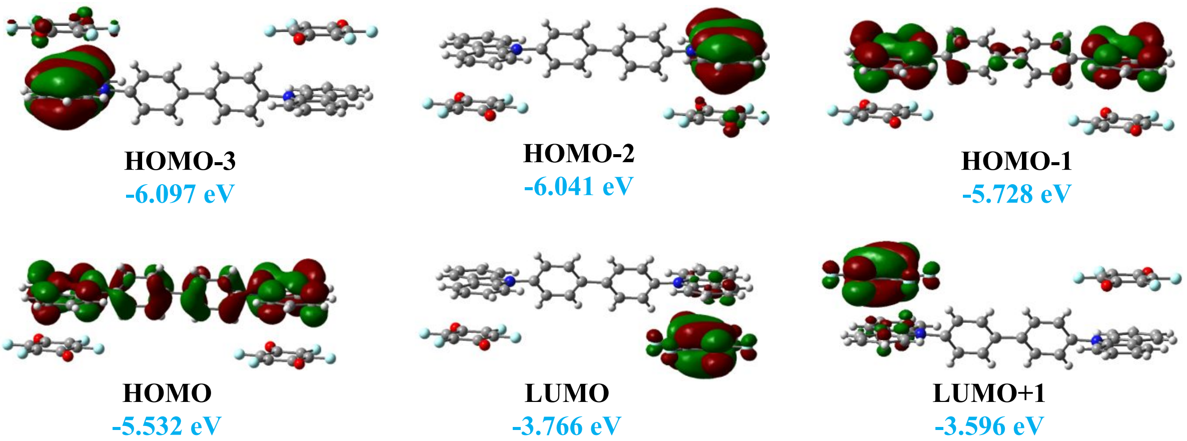

Figure S4. The frontier molecular orbital of π-stacked CBP:(fluoranil)$_2$ trimer, calculated at Grimme's dispersion corrected B3LYP-D3/6-31G(d,p) level of theory.

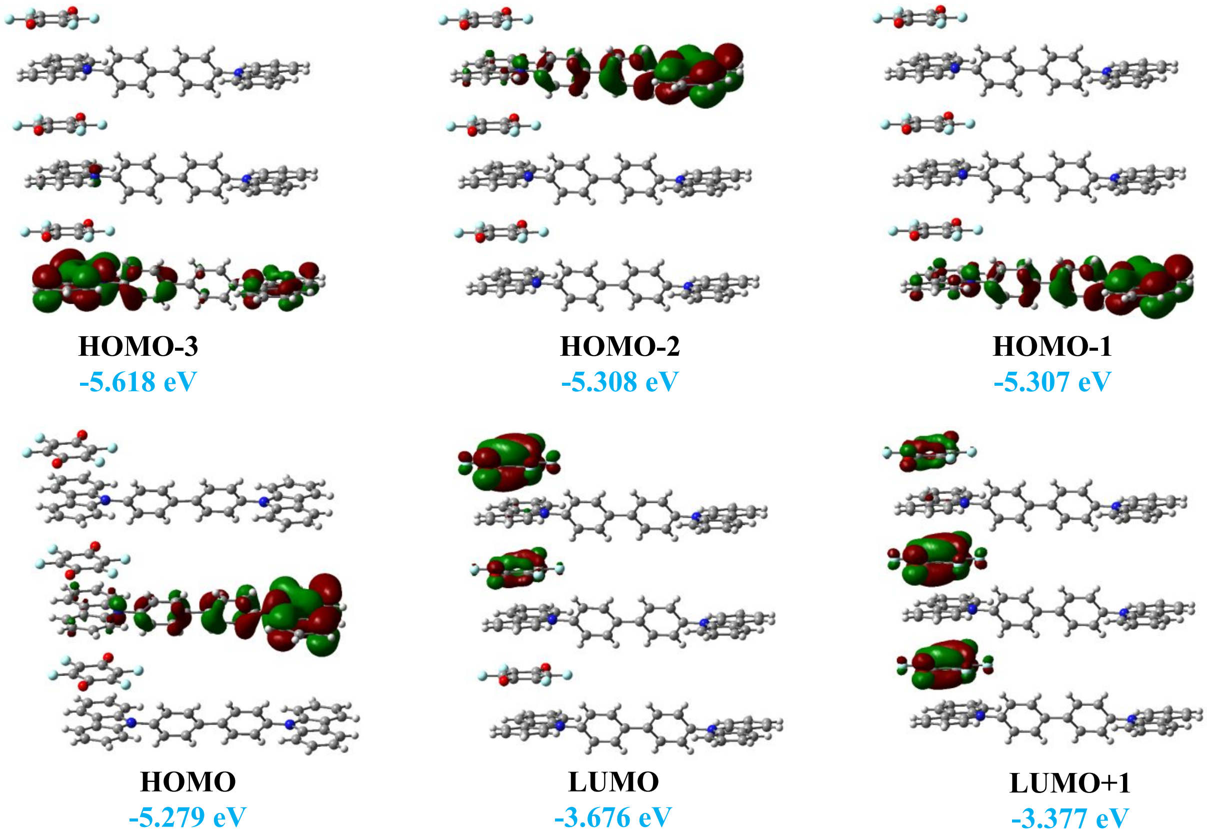

Figure S5. The frontier molecular orbital of π-stacked D–A–D–A–D–A hexamer, calculated at dispersion corrected B3LYP-D3/6-31G(d,p) level of theory.

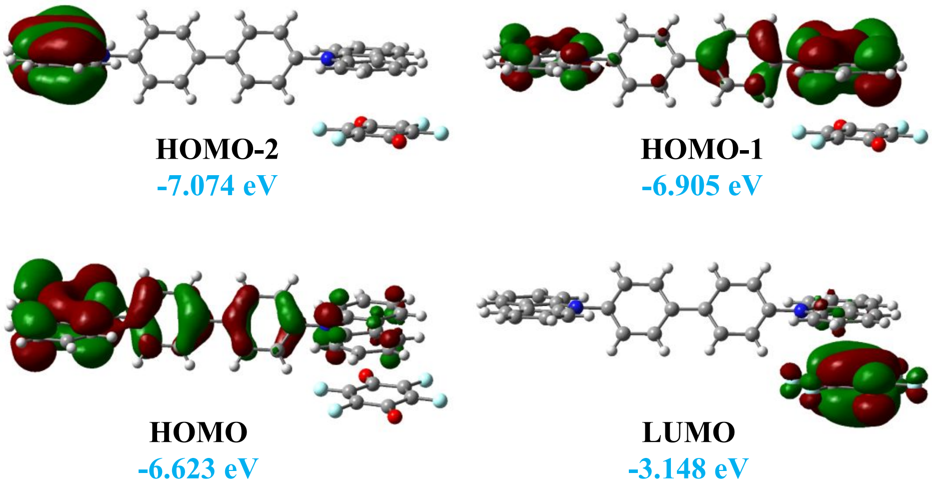

Figure S6. The frontier molecular orbital of π-stacked D–A dimer, calculated at CAM-B3LYP/6-31G(d,p) level of theory.

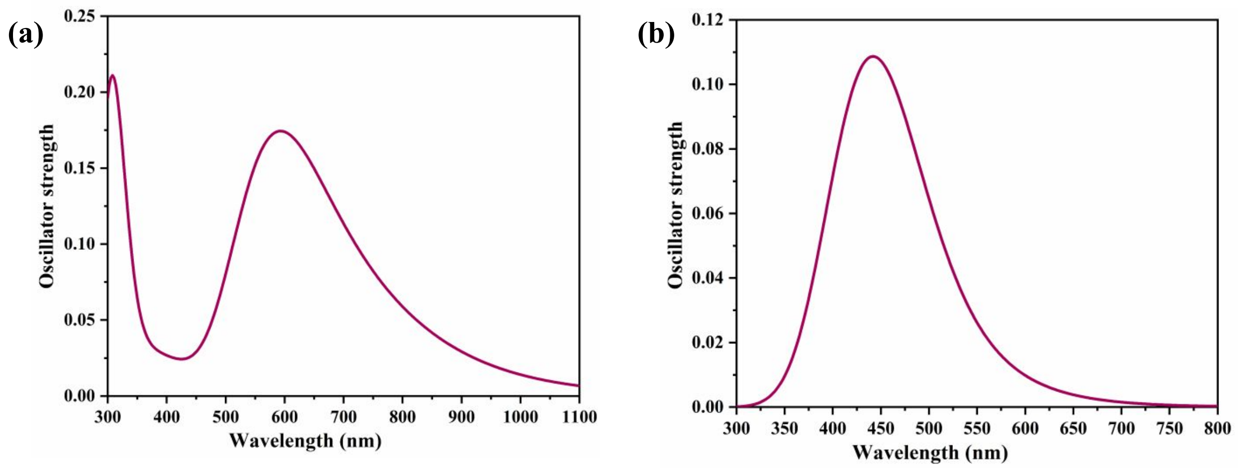

Figure S7. (a) Calculated absorption spectrum from time dependent DFT calculation using coordinates of π-stacked D–A dimer at CAM-B3LYP/6-31G(d,p) level of theory; (b) calculated absorption from time dependent DFT calculation spectrum using π-stacked D–A–D–A dimer at CAM-B3LYP/6-31G(d,p) level of theory.

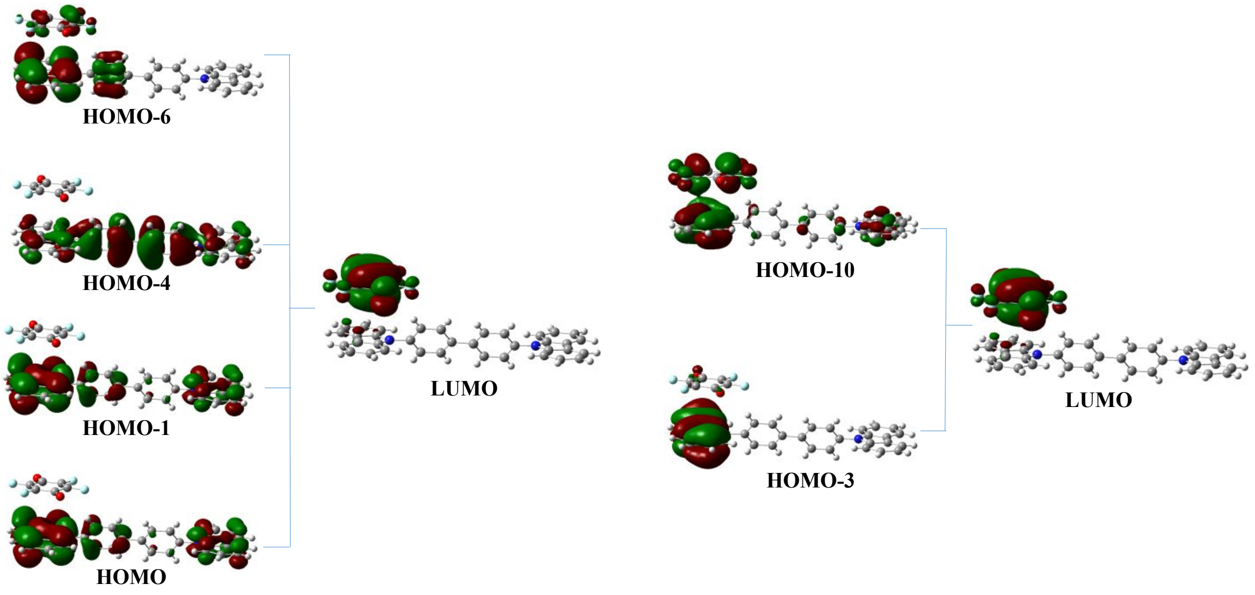

Figure S8. Molecular orbitals taking part in constructing S_1-S_2 excited states in the π-stacked CBP:fluoranil dimer, time dependent DFT calculation at ω97X-D/6-31G(d,p) level of theory.

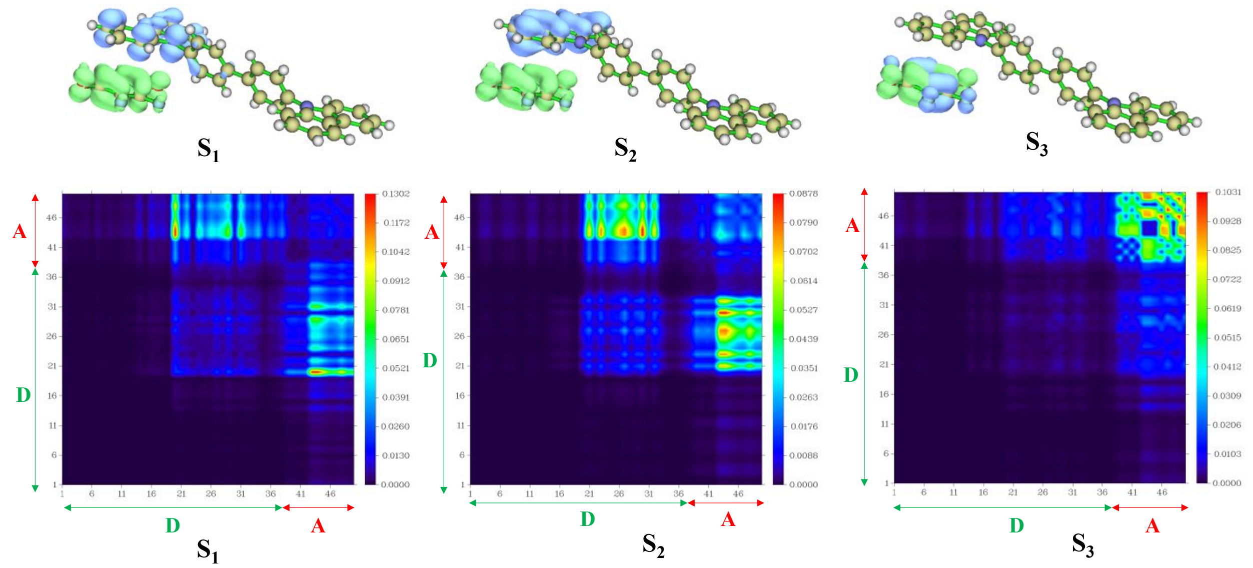

Figure S9. (a) Electron (green) and hole (blue) distribution in the S$_1$-S$_2$ excited states of π-stacked CBP:fluoranil dimer calculated at ω97X-D/6-31G(d, p) level and plotted at iso-surface 0.002 a.u.; (b) one electron transition density matrix (TDM) heat maps for the S$_0$→S$_1$, S$_0$→S$_2$ and S$_0$→S$_3$ transitions in the π-stacked D–A dimer, the off-diagonal elements in heat maps of S$_0$→S$_1$ and S$_0$→S$_2$ indicate charge transfer nature of the excitations, while the S$_0$→S$_3$ transition is characterized with prominent diagonal element.

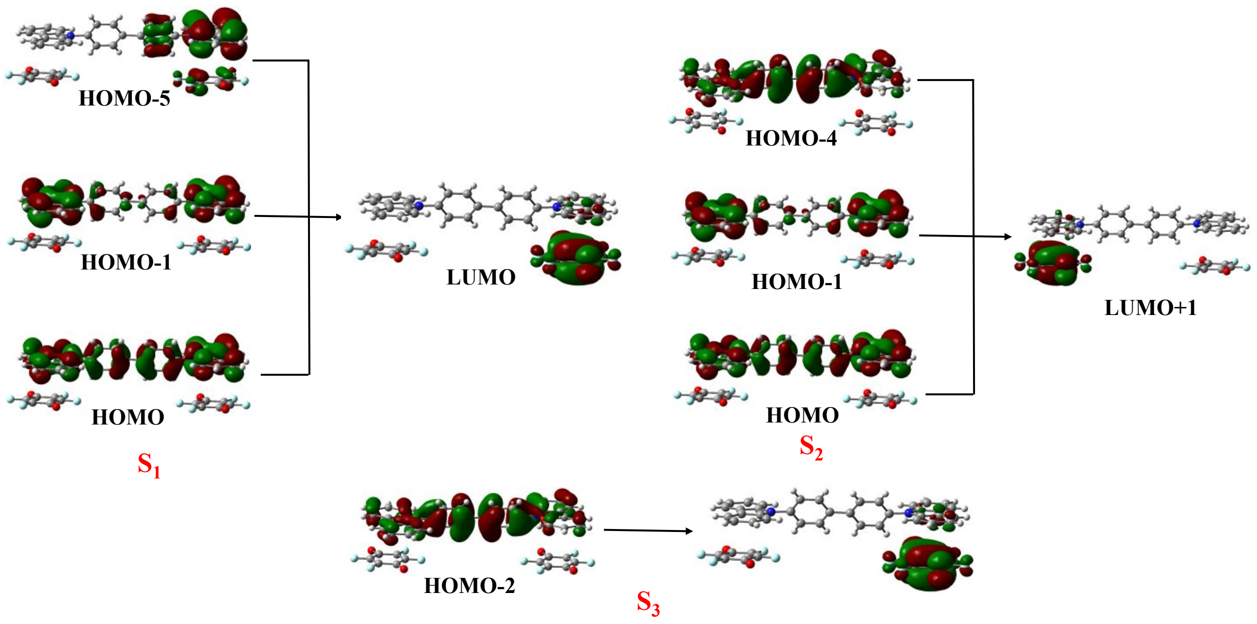

Figure S10. Molecular orbitals taking part in constructing S_1-S_3 excited states in CBP:(fluoranil)$_2$ trimer, time dependent DFT calculation at CAM-B3LYP/6-31G(d,p) level of theory.

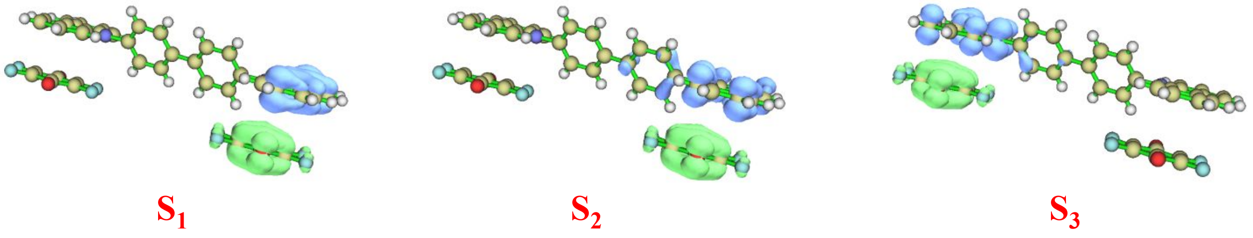

Figure S11. Electron-hole distribution in excited S_1-S_3 excited states in CBP:(fluoranil)$_2$ trimer, calculated at CAM-B3LYP/6-31G(d,p) level of theory. Electrons are shown in blue and holes are shown in green, drawn at iso-density 0.002 a. u.

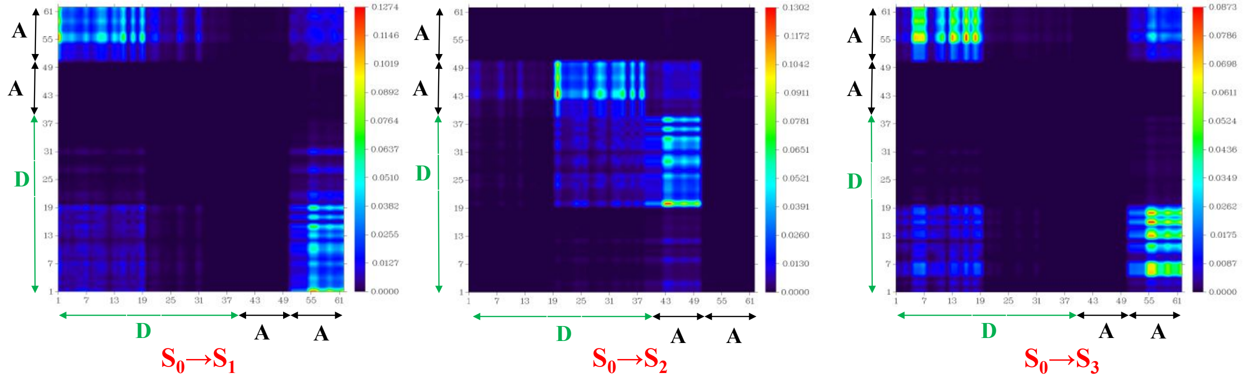

Figure S12. Heat maps for one electron transition density matrix in excited S_1-S_3 excited states in CBP:(fluoranil)$_2$ trimer, calculated at CAM-B3LYP/6-31G(d,p) level of theory. Prominent off-diagonal elements and the absence of any distinct diagonal elements indicate charge transfer origin of the excited states.

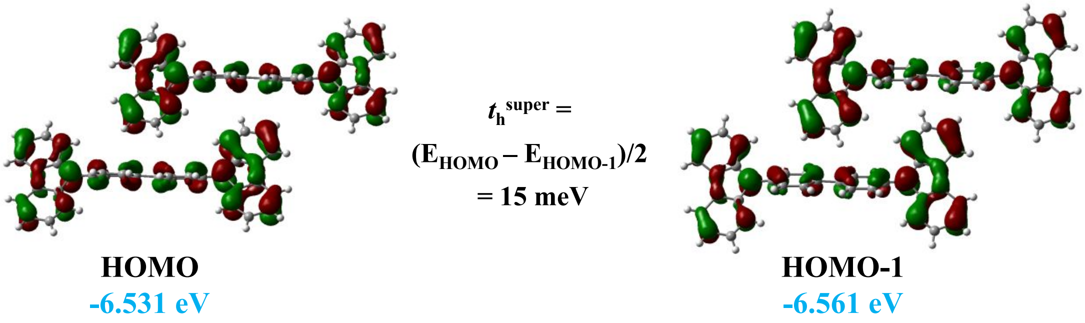

Figure S13. Direct hole transfer integral calculated from CBP dimer, calculation at CAM-B3LYP/6-31G(d,p) level of theory.

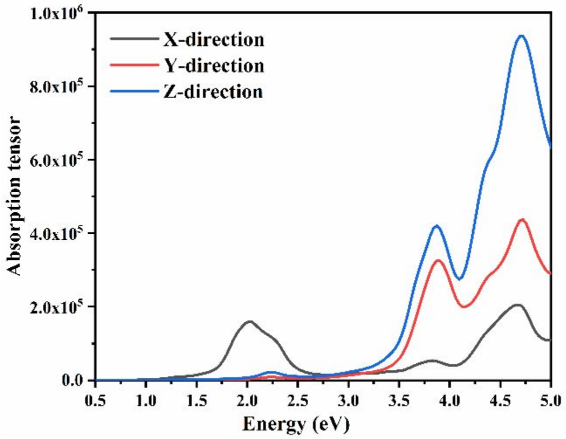

Figure S14. Calculated absorption spectrum using unit cell geometry at 100 K, HSE06 functional and a Γ-centered 3×3×1 mesh. It shows that the light absorption in the visible and NIR will be maximum along the x-direction.
